# Genetic Analysis of Mps3 SUN Domain Mutants in *Saccharomyces cerevisiae* Reveals an Interaction with the SUN-Like Protein Slp1

**DOI:** 10.1534/g3.112.004614

**Published:** 2012-12-01

**Authors:** Jennifer M. Friederichs, Jennifer M. Gardner, Christine J. Smoyer, Christine R. Whetstine, Madelaine Gogol, Brian D. Slaughter, Sue L. Jaspersen

**Affiliations:** * Stowers Institute for Medical Research, Kansas City, Missouri 64110, and; † Department of Molecular and Integrative Physiology, University of Kansas Medical Center, Kansas City, Kansas 66160

**Keywords:** Mps3, SUN protein, spindle pole body duplication, nuclear/ER membrane, SGA

## Abstract

In virtually all eukaryotic cells, protein bridges formed by the conserved inner nuclear membrane SUN (for Sad1-UNC-84) domain-containing proteins and their outer nuclear membrane binding partners span the nuclear envelope (NE) to connect the nucleoplasm and cytoplasm. These linkages are important for chromosome movements within the nucleus during meiotic prophase and are essential for nuclear migration and centrosome attachment to the NE. In *Saccharomyces cerevisiae*, *MPS3* encodes the sole SUN protein. Deletion of *MPS3* or the conserved SUN domain is lethal in three different genetic backgrounds. Mutations in the SUN domain result in defects in duplication of the spindle pole body, the yeast centrosome-equivalent organelle. A genome-wide screen for mutants that exhibited synthetic fitness defects in combination with *mps3* SUN domain mutants yielded a large number of hits in components of the spindle apparatus and the spindle checkpoint. Mutants in lipid metabolic processes and membrane organization also exacerbated the growth defects of *mps3* SUN domain mutants, pointing to a role for Mps3 in nuclear membrane organization. Deletion of *SLP1* or *YER140W/EMP65* (for ER membrane protein of 65 kDa) aggravated growth of *mps3* SUN domain mutants. Slp1 and Emp65 form an ER-membrane associated protein complex that is not required directly for spindle pole body duplication or spindle assembly. Rather, Slp1 is involved in Mps3 localization to the NE.

The nuclear envelope (NE) of eukaryotic cells has evolved to organize and protect the genome. The double lipid bilayer of the NE is composed of an outer nuclear membrane (ONM) that is contiguous with the endoplasmic reticulum (ER) and contains many of the same proteins and an inner nuclear membrane (INM) that is composed of a distinct set of proteins, some of which directly interact with chromosomes within the nucleus to control gene expression and DNA recombination and repair (Schirmer and Gerace 2005). Nuclear pore complexes (NPCs) are present at sites in the NE in which the INM and ONM are joined to form a third NE membrane domain, the pore membrane (Hetzer and Wente 2009). NPCs play a central role in communication between the cytoplasm and the nucleoplasm, including import of transcription factors, histones, and signaling molecules; export of all mRNAs; and import and export of proteins and RNAs involved in ribosome assembly, splicing, and other vital cellular processes (Weis 2003). A series of partially redundant karyopherins and the conserved GTP-binding protein Ran facilitate NPC-mediated transport of cargos greater than approximately 30−40 kDa into and out of the nucleus of all eukaryotes (Terry *et al.* 2007).

In addition to communication via the NPC, eukaryotic cells have evolved at least two more pathways for nuclear−cytoplasmic interaction. One pathway involves the budding and fusion of vesicles from the INM to ONM, which was recently shown to deliver mRNP particles from the nucleus to cytoplasm of neuronal cells (Speese *et al.* 2012). This is similar to the nuclear-cytoplasmic trafficking mechanism used by certain types of viruses (Mettenleiter *et al.* 2006). A second pathway of nuclear−cytoplasmic interaction involves a linker complex that spans the lumenal space between the INM and ONM, coupling the nucleoskeleton or chromatin with the cytoplasmic cytoskeleton. Known as the LINC complex, for linker of nucleoskeleton and cytoskeleton, a bridge is formed by association of the highly conserved SUN protein (for Sad1-UNC-84 homology domain) localized to the INM and an ONM partner, which frequently, but not always, contains a C-terminal KASH domain [for Klarsicht-Anc-1-Syne-1 homology (Razafsky and Hodzic 2009; Starr and Fridolfsson 2010)]. Studies from multiple eukaryotes have shown roles for SUN and KASH proteins in meiotic chromosome movements, nuclear migration and positioning, centrosome function, regulation of gene expression, and DNA double-stand break repair (Burke and Roux 2009; Hiraoka and Dernburg 2009; Morimoto *et al.* 2012; Razafsky and Hodzic 2009; Starr and Fridolfsson 2010).

SUN-domain containing proteins contain three structural features: a transmembrane, a coiled-coil, and a SUN domain. At least one transmembrane domain is responsible for anchoring SUN proteins in the INM so that the N-terminal region is oriented toward the nucleoplasm and the larger C-terminal domain is present in the lumenal space between the INM and ONM (Malone *et al.* 1999; Starr and Fridolfsson 2010; Starr and Han 2002; Wilson and Dawson 2011). Some SUN proteins such as *Schizosacchromyces pombe* Sad1, *Saccharomyces cerevisiae* Mps3, and mouse Sun2 contain chromatin-binding motifs in their N-terminal domains, which facilitate interactions with chromosomes (Bupp *et al.* 2007; Chikashige *et al.* 2006; Jaspersen *et al.* 2006; Lei *et al.* 2012; Miki *et al.* 2004; Tang *et al.* 2006). SUN proteins in other organisms such as *Caenohabditis elegans* lack these chromatin-binding motifs, but there is substantial evidence that these SUN proteins at least indirectly associate with DNA-binding elements such as the meiotic pairing center proteins (Hiraoka and Dernburg 2009; Jaspersen and Hawley 2011). Sun1 also associates with telomeres during gametogenesis in mice, although the molecules that mediate this interaction have not been elucidated (Ding *et al.* 2007).

The larger C-terminal region of SUN proteins contains at least one coiled-coil domain, which is thought to play a role in oligomerization of SUN proteins (Crisp *et al.* 2006; Ostlund *et al.* 2009; Wang *et al.* 2006). Studies of recombinant Sun2 binding to KASH domain peptides showed a requirement for the coiled-coil region, as well as the SUN domain, in binding to the KASH motif (Sosa *et al.* 2012; Wang *et al.* 2012; Zhou *et al.* 2012). This result is somewhat surprising based on data from *S. cerevisiae* demonstrating that the coiled-coil region of Mps3 is nonessential for vegetative growth and sporulation (Friederichs *et al.* 2011; Lee *et al.* 2012). One explanation for this discrepancy is that budding yeast may lack *bona fide* KASH proteins. Because of this, the interaction of Mps3 with proteins via its C-terminal SUN domain may occur in a manner that is distinct from other SUN-domain containing proteins. Another possibility is that additional factors mediate the interaction between SUN and KASH proteins *in vivo* such that the coiled-coil region is less critical than when binding is assayed *in vitro*.

The third conserved feature of SUN proteins is the SUN domain itself. This domain is almost always located at the C-terminus of the protein and is found in most eukaryotes (Malone *et al.* 1999; Starr and Fridolfsson 2010; Starr and Han 2002; Wilson and Dawson 2011). Recent structural studies on the SUN domain derived from mammalian Sun2 indicated that this region folds into homotrimeric structure, primarily composed of a series of β-sheets that resembles the sugar-binding region found in lectins, suggesting that these proteins share an ancient common ancestor (Burke 2012; Sosa *et al.* 2012). On the basis of the crystal structure, key residues that mediate interactions between adjacent SUN domains and between SUN-KASH domains were predicted and many were found to be important for Sun2-Nespirin-2 binding *in vitro* (Sosa *et al.* 2012; Wang *et al.* 2012; Zhou *et al.* 2012). However, the role of some residues in SUN-KASH binding was ambiguous in these analyses, perhaps because additional factors that modulate the formation of the LINC complex *in vivo* were absent or features of the SUN-KASH complex were not accurately represented in the crystal structures.

*MPS3* encodes the sole SUN protein in budding yeast (Jaspersen *et al.* 2006). Although Mps3 is not known to bind to a KASH domain-containing protein and it lacks many of the residues thought to be critical for SUN-KASH binding based on the crystallographic studies, previous mutagenesis of the Mps3 SUN domain showed that key conserved residues were critical for Mps3 function in spindle pole body (SPB) duplication, nuclear migration after mating (known as karyogamy) and binding to the membrane-associated SPB component Mps2 within the lumenal space (Antoniacci *et al.* 2004; Jaspersen *et al.* 2002, 2006; Nishikawa *et al.* 2003). This finding suggests a basic mechanism of SUN protein action is conserved in all eukaryotic SUN proteins, allowing for the analysis of additional factors that influence SUN protein function and binding within the lumenal space through genetic analysis of *MPS3*.

In the current work, we have examined the function of the Mps3 SUN domain in multiple yeast backgrounds and show that it is essential for viability during both vegetative growth and sporulation. Genetic analysis of mutants in the Mps3 SUN domain revealed a role in mitotic spindle formation, consistent with previous analyses of these mutants (Jaspersen *et al.* 2006). In addition, our studies predict a role for Mps3 in other processes such as chromatin structure and lipid metabolism. We also find that mutations in the Mps3 SUN domain display enhanced growth defects with deletions in the gene encoding the SUN-like protein, *SLP1*, and an uncharacterized open reading frame (ORF) YER140W, which we have named *EMP65* (for ER membrane protein of 65 kDa). We show that Slp1 and Emp65 form an ER-associated membrane complex. Slp1 and Emp65 are not required for SPB duplication or mitotic spindle assembly. However, Slp1 is required for efficient Mps3 localization to the INM, suggesting at least an indirect link to Mps3 function at the NE.

## Materials and Methods

### Yeast strains and plasmids

All strains are listed in supporting information, Table S2. The yeast deletion collection was purchased from Open Biosystems. Standard techniques were used for DNA and yeast manipulations.

Deletion of and tagging of *MPS3*, *SLP1*, *EMP65*, *TUB4*, and *HTB2* was done by polymerase chain reaction (PCR)-based methods (Longtine *et al.* 1998; Sheff and Thorn 2004) and was verified by PCR. YIplac204-HDEL-DsRed (a gift of Ben Glick, University of Chicago) was integrated into *TRP1* after digestion with *Eco*RV. To visualize microtubules, *HIS3pr-mCherry-TUB1* from pLH30 (a gift of Soni Lacefield, University of Indiana) was subcloned into pRS304 at *Kpn*I-*Not*I. The HYGMX marker expressed from *AgTEF1pr* was amplified by PCR and inserted into *Not*I-*Sac*I to create pSJ1372. Digestion with Bsu36I directed integration into *TRP1*, which is intact in the BY background, and integration was selected using Hyg^R^.

Construction of pSJ148 (pRS305-*MPS3*) and mutants in the Mps3 SUN domain has been previously described (Jaspersen *et al.* 2006). Plasmids were digested with *Bst*EII to target integration to *LEU2*, and the number of copies of *MPS3* integrated was determined by Southern blotting. To integrate alleles at the *MPS3* locus, the mutant *mps3* gene was amplified using OSJ421 (atgaataactcaaatgagcatag) and OSJ423 (cgccagtaccgaaagagagtggtgagtagctgattaactctatgatttaaagggcagtatagcgaccagcattcac) and NATMX was amplified using OSJ420 (acatggaggcccagaataccctcctt) and OSJ422 (cgccagtaccgaaagagagtggtgagtagctgattaactctatgatttaaagggcagtatagcgaccagcattcac). Both PCR products were cotransformed into yeast, and integration of the mutant allele was confirmed by PCR analysis and sequencing.

For dilution assays, 5 OD_600_ of cells were serially-diluted 10-fold in sterile growth media and stamped onto agar plates. YPD has 2% glucose, YPGR has 2% galactose and 2% raffinose, and YPEG has 2% ethanol and 2% glycerol as the carbon source. Chemicals were purchased from Sigma-Aldrich and were added to media in the following amounts: 5 mM oleic acid, 0.2% benzyl alcohol, 5 µg/mL terbinafine, 10 µg/mL benomyl, 0.5 µg/mL tunicamycin, 2 mM DTT, 450 µM fumonisin, 0.5 µg/mL, 1 µg/mL ketoconazole, 100 µg/mL clonNAT, 200 µg/mL G418, 50 µg/mL canavanine, and 50 µg/mL thialysine. Media for SGA was prepared as described (Tong and Boone 2006).

### Synthetic genetic array screen

The SGA screen was performed essentially as previously described (Tong and Boone 2006) using the Singer RoToR robot. Because our query strains contained a *pURA3-MPS3* plasmid to prevent spontaneous diploidization, after mating of the query (*α mps3*::mps3*-NATMX can1Δ::*STE2pr-HIS3MX lyp1Δ ura3his3 pURA3-MPS3*) to the deletion collection (*a yfgΔ*::*KANMX LYP1CAN1his3ura3*) and diploid selection on YPD+G418+clonNAT, cells were pinned to 5-fluoroorotic acid (5-FOA) to select for loss of the covering plasmid. Diploids were sporulated for 3−4 weeks then haploids were selected twice on SD-His-Lys-Arg+thialysine+canavanine before pinning to SD/MSG-His-Lys-Arg+thialysine+canavanine+G418 and SD/MSG-His-Lys-Arg+thialysine+canavanine+G418+clonNAT. All plates were incubated at 23°.

Each screen was done in triplicate, and a list of 400 potential interacting genes was identified based on visual inspection of the plates. A mini-library containing these 400 deletion mutants was crossed to all four *mps3* mutants in a second round of screening, with each deletion present in quadruplicate. A list of potential hits was generated based on a synthetic growth defect in at least three of the four colonies in at least two of the three mini-screens performed. These hits were further verified by random sporulation and/or tetrad dissection. Random sporulation was performed by resuspending a sporulated colony from the 1536-well plate in 500 µL of sterile water. After vortexing to disperse the cells, 200 µl of cells was plated to SD/MSG-his-arg-lys+thialysine+canavanine+G418+NAT and 100 µL of cells was plated onto SD/MSG-his-arg-lys+thialysine+canavanine+G418 and SD/MSG-his-arg-lys+thialysine+canavanine+NAT plates. Then, 50 µL of cells was added to 150 µL of water and all 200 µL was plated onto SD-his-arg-lys+thialysine+canavanine. Plates were incubated at 23°. In total, 106 genetic interactions were identified and verified in this screen. ORFs, their corresponding gene, and the strength of the genetic interaction are summarized in Table S1.

Genetic interaction data were downloaded from BioGRID 3.1.88, and interacting gene pairs that were curated as synthetic lethal, synthetic growth defect, and negative genetic interaction were extracted. Data from our study were integrated to create a 0−1 matrix that contains 4118 ORFs. In this matrix, 1 represents a negative interaction or synthetic growth/lethal defect between two gene pairs whereas gene pairs that do not interact are represented with 0. Jaccard distance was used to measure the similarity of our *mps3* mutant profiles and the similarity between *mps3* mutants and other mutants in the BioGRID dataset (Jay *et al.* 2012). A gene with a distance of less than 0.95 to the query gene (one of our *mps3* mutants) was considered to have a similar genetic signature since the majority (95%) of budding yeast ORFs in BioGRID fall above this cut off (data not shown). 296 genes had a Jaccard distance <0.95 to one or more of our *mps3* mutants and 32 had a Jaccard distance of <0.95 to all three *mps3* SUN domain mutants. GO analysis and two-dimensional hierarchical clustering of these genes were performed as previously described (Tong *et al.* 2001, 2004).

### Cytological techniques

Fluorescence imaging of Slp1-3xGFP, GFP-Emp65, Mps3-GFP, H2B-mCherry, and HDEL-dsRed was performed using a confocal microscope (LSM-510-META; Zeiss, Inc) equipped with a ConforCor 3 module with avalanche photodiode detectors, which allow single photon counting, with 63X 1.4 NA Plan-Apochromat or 100X 1.46 NA α-Plan Fluar objectives (Zeiss, Inc). GFP and mCherry or dsRed were excited using the 488-nm and 561-nm laser lines, respectively. Emitted photons were collected through BP 505-540 nm and LP 580-nm filters, with a pinhole size of 1.03 Airy units according to the green channel. Data were acquired using AIM v.4.2 software (Zeiss, Inc). Images were collected with 8−10 image stacks with a 0.3-μm step size through the cells at room temperature. Images were processed using ImageJ software (NIH). At least two independent transformants of each genotype were analyzed by fluorescence microscopy in at least three independent experiments.

The distribution of Mps3-GFP to the SPB and NE was quantitated as previously described (Gardner *et al.* 2011). We created a mask based on the transmitted light image of cells using Axiovision software (Zeiss, Inc.). Next, we selected all pixels above a predefined threshold of the H2B-mCherry signal to create an additional mask that describes the nucleus. The outer pixels of this mask are considered the NE and are used to quantify the amount of Mps3-GFP localized in the NE (INM signal) compared with the amount protein that is mislocalized (non-NE/ER signal = nonNE). We can then determine a ratio of mean intensity per unit area for each. The very bright SPB signal from Mps3-GFP is excluded from this calculation using an upper intensity cut-off. At least 30 images of each genotype were quantified per experiment, and NE/nonNE ratio for each was plotted using OriginPro. Outliers of greater than 2 SDs were excluded from analysis. To generate Figure 8A, images were spatially binned 2 by 2, then slices that represent the center of the NE were selected. A max projection was performed over these slices, and the final image was spatially smoothed in ImageJ (NIH).

Microtubules and SPBs were visualized in *mCherry-TUB1* and *TUB4-GFP* expressing cells using a 100× 1.4 NA oil objective on an inverted Zeiss 200m equipped with a Yokagawa CSU-10 spinning disc. Then, 488-nm excitation and 568-nm excitation were used for GFP and mCherry, respectively, and emission was collected through BP 500- to 550-nm and BP 590- to 650-nm filters, respectively, onto a Hamamatsu EMCCD (C9000-13). For each channel, a Z-stack was acquired using 0.6 or 0.7 μm spacing. A total of 13 total slices were acquired, and a max projection image was created for analysis of foci using ImageJ (NIH) to calculate the distance between SPBs in large budded cells. Analysis of DNA content by flow cytometry and DAPI staining were performed as previously described (Jaspersen *et al.* 2002).

### Immunoprecipitation, western blotting, and Pro-Q Emerald staining

Liquid nitrogen ground lysates were prepared from mid-log phase cells as described (Bupp *et al.* 2007; Jaspersen *et al.* 2006). To summarize, cells were frozen in liquid nitrogen and ground with a Retsch ball mill. Ground cell powder was allowed to thaw on ice then resuspended in 9 mL of extraction buffer (20 mM Hepes-NaOH, pH 7.5; 300 mM NaCl; 1 mM EDTA; 5 mM EGTA; 50mM NaF; 50 mM β-glycerophosphate; 0.5% TritonX-100; 1 mM DTT; 1 mM PMSF; and 1 mg/mL each pepstatin A, aprotinin, and leupeptin). Following homogenization with a Polytron 10/35 for 30 sec, lysates were centrifuged at 3000 × g for 10 min. at 4° and the resulting supernatant was used for immunoprecipitations. 100 µl anti-HA resin (Roche) or 100 µl of anti-FLAG resin (Sigma) was added to lysates to immunoprecipitate tagged proteins. After a 2-hr incubation at 4°, beads were washed 5 times and 1/10^th^ of the bound protein and 1/50^th^-1/100^th^ of the input lysate was analyzed by sodium dodecyl sulfate-polyacrylamide gel electrophoresis (SDS-PAGE) followed by western blotting. In all figures, positions of molecular weight markers (kDa) are indicated next to each blot, and asterisks mark the position of cross-reacting bands present in the control sample.

The following primary antibody dilutions were used: 1:1000 anti-HA 3F10 (Roche), 1:1000 anti-FLAG M2 (Sigma-Aldrich), 1:2000 anti-G6PDH (Sigma-Aldrich), and 1:1000 anti-GFP (Roche). Alkaline phosphatase conjugated secondary antibodies were used at 1:10000 (Promega), and fluorescently conjugated secondary antibodies were used at 1:10000 for analysis and quantification using the Odyssey system (Li-Cor).

Carbohydrates were detected on immunoprecipitated proteins after SDS-PAGE using the Pro-Q Emerald staining kit from Life Technologies according to the manufacturer’s instructions. Silver staining was performed using the SilverXpress silver staining kit from Life Technologies.

## RESULTS

### Characterization of *mps3* mutants

**Figure 1:**
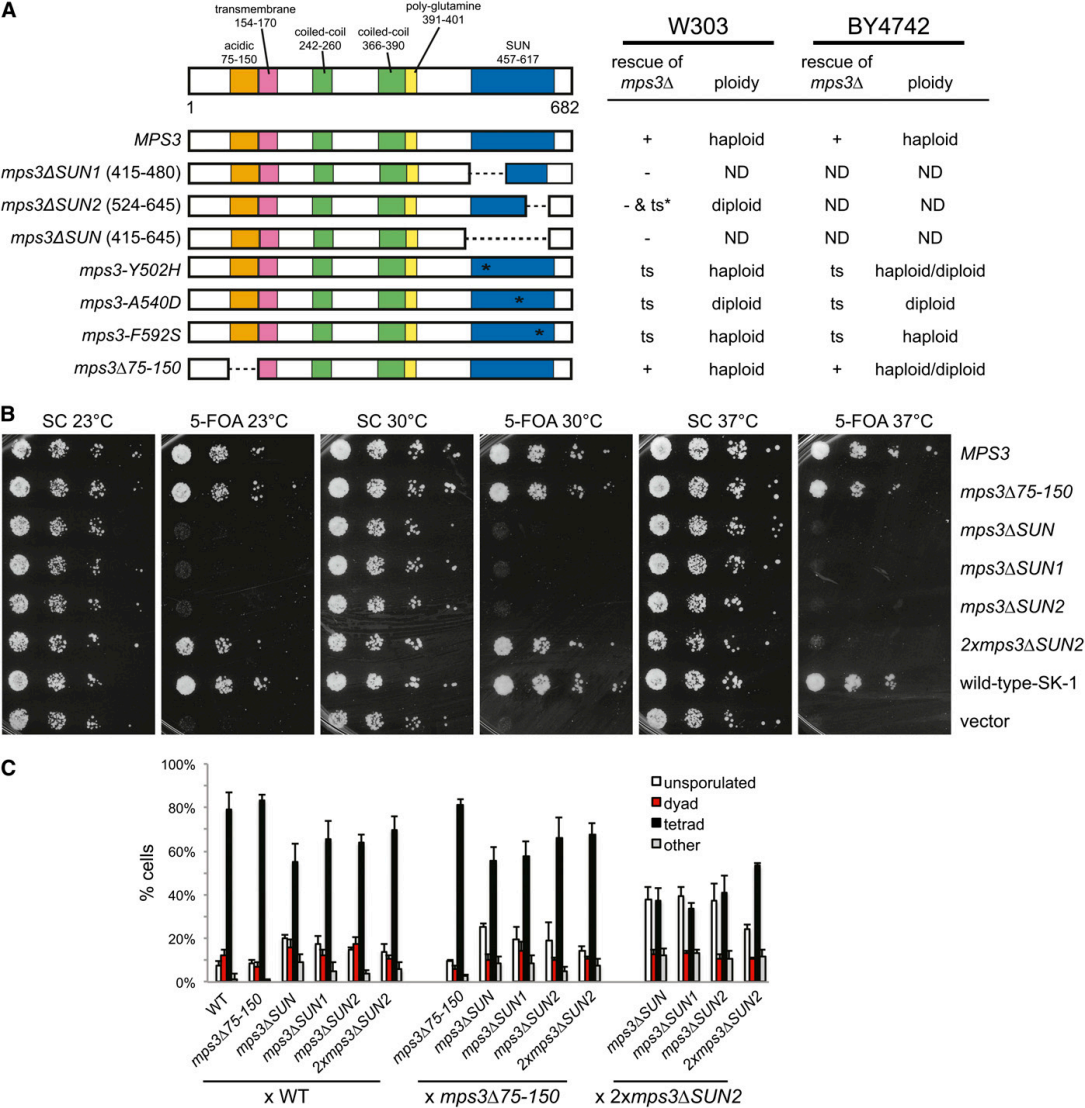
Effects of mutation or deletion of the *MPS3* SUN domain. (A) Schematic of Mps3 showing the amino acid positions of the acidic, transmembrane, coiled-coil, poly-glutamine, and SUN domains. Below are versions of wild-type *MPS3* and various mutants in the SUN domain, including *mps3ΔSUN*, *mps3ΔSUN1*, and *mps3ΔSUN2* that lack the indicated residues; three SUN domain mutants, *mps3-Y502H*, *mps3-A540D*, and *mps3-F592S*; and a mutant in the Mps3 N-terminus, *mps3Δ75-150*, which lacks the acidic domain. The ability of each version of *MPS3* to rescue a W303-based *mps3Δ* (SLJ1053) was previously determined (Jaspersen *et al.* 2006), and the ability of each to rescue a BY4742-based *mps3Δ* (SLJ4186) was tested. + indicates the allele rescues growth at all temperatures; – indicates the allele is unable to rescue growth at any temperature and ts indicates the allele is able to rescue growth at 23° but not at 37°. For *mps3ΔSUN2*, rescue depended on the number of copies of the mutant allele present. The ploidy of viable strains is shown. (B) The ability of *MPS3* and other alleles to rescue growth of *mps3Δ* in SK-1 (SLJ6406) was tested by plating 10-fold serial dilutions of homozygous diploid cells onto SC-complete or 5-FOA. Plates were incubated for 2 d at 30° and 37° and for 3 d at 23°. (C) Diploid SK-1 strains of the indicated genotypes were grown overnight at 23° in YPEG followed by YPD for 24 hr before they were transferred to sporulation media for 72 hr at 23°. Meiotic progression was analyzed based on DAPI staining and DIC images. The percentage of unsporulated cells, dyads (two DNA masses in two spores per asci), and tetrads (four DNA masses in four spores per asci) was determined (n = 200 in three independent experiment; error bars, SEM). In some cases, more than four spores or DNA masses, or multiple DNA masses in a single cell/spore were observed; these were classified as “other.”

Previously, we showed that the Mps3 SUN domain was essential (Jaspersen *et al.* 2006). Yeast cells containing a complete or partial deletion of the SUN domain were inviable (Figure 1A). Recently, it was proposed that the Mps3 SUN domain was not essential for viability in the SK-1 strain background, possibly due to the lack of a factor that restricts Mps3 function to the SPB (Rao *et al.* 2011). We integrated our alleles in the Mps3 SUN domain (see Figure S1) into an SK-1−derived strain as well as into a BY-derived strain (an S288c derivative) containing a deletion of *MPS3* at the genomic locus covered by a wild-type copy of *MPS3* on a *URA3*-based centromeric plasmid. The ability of each allele to serve as the sole copy of *MPS3* was tested by growing cells on 5-FOA, which selects for cells that have lost the wild-type p*URA3-MPS3* plasmid. We found that deletion of the entire SUN domain (*mps3ΔSUN*, amino acids 415-645) or removal of its first (*mps3ΔSUN1*, amino acids 415-480) or second half (*mps3ΔSUN2*, amino acids 524-645) are lethal in both SK-1 and BY strain backgrounds. However, if two copies of the *mps3ΔSUN2* allele are present (*2xmps3ΔSUN2*), the strain is temperature-sensitive (ts; Figure 1, A and B). These phenotypes are virtually identical to those previously observed in W303 (Jaspersen *et al.* 2006).

Using the SK-1 derived alleles of *MPS3*, we analyzed the requirement for the SUN domain during meiosis by analyzing the formation of spores in heterozygous and homozygous diploids. At 72 hr after transfer to media lacking nitrogen in the presence of limiting levels of carbon, we found that strains containing a wild-type copy of *MPS3* and any of the *mps3* SUN domain mutants were able to complete the meiotic program and form viable progeny in approximately 50–80% of cells (Figure 1C). In the most severe case, only 55 ± 8% of *MPS3/mps3ΔSUN* heterozygotes formed asci containing four spores compared with 79 ± 7% in *MPS3/MPS3*. Dissection of 20 tetrads formed in *MPS3/mps3ΔSUN* heterozygotes showed two viable spores and two inviable spores. The inviability is presumably due to the requirement for the Mps3 SUN domain during vegetative growth since all of the viable spores contained markers associated with the wild-type copy of *MPS3* but not the *mps3ΔSUN* mutant allele. If *MPS3/2xmps3ΔSUN2* tetrads were analyzed, four viable progeny were observed in 16/20 tetrads, suggesting that a single functional copy of the SUN domain is sufficient for successful completion of meiosis and spore formation.

Because *mps3ΔSUN/mps3ΔSUN* is not viable during the mitotic divisions leading up to the initiation of meiosis, we could not examine the effect of the homozygous SUN domain deletion on spore formation. However, we could examine the phenotype associated with deletion of the second half of the SUN domain because *2xmps3ΔSUN2/2xmps3ΔSUN2* cells are viable, at least at 23°. In *2xmps3ΔSUN2/2xmps3ΔSUN2* homozygotes, 53 ± 1% of cells formed four-spore tetrads (Figure 1C). When *2xmps3ΔSUN2* was combined with other deletions in the SUN domain, we observed profound defects in spore formation, including an increase in unsporulated cells and the production of dyads (Figure 1C). Unlike the progeny from crosses to cells containing a wild-type copy of *MPS3*, the four-spore tetrads formed during these meiotic divisions were generally inviable; *2xmps3ΔSUN2/2xmps3ΔSUN2* homozygotes resulted in four-viable progeny in 4 of 20 tetrads, and *2xmps3ΔSUN2/mps3ΔSUN* never resulted in viable progeny in the 20 tetrads analyzed. This suggests that there is a severe defect(s) in some aspect of the meiotic program such as pairing, recombination, chromosome segregation or spore formation in the absence of the Mps3 SUN domain. When the N-terminal region between amino acids 75−150 was deleted, we saw little effect on meiotic progression and spore formation, consistent with previous reports (Lee *et al.* 2012). Heterozygotes containing this allele and an *mps3* SUN allele did not show enhanced effects on spore formation (Figure 1C). Therefore, we conclude that the Mps3 SUN domain is essential during both mitotic growth and sporulation.

Mutations in conserved residues within the SUN domain also frequently resulted in nonfunctional versions of *MPS3* (Jaspersen *et al.* 2006); however, some mutations were at least partially functional in all three strain backgrounds, resulting in ts alleles of *MPS3* (Figure 1A, Figure S1, and data not shown). Several phenotypes associated with these mutants suggested a defect in SPB duplication. First, flow cytometric analysis of DNA content showed that *mps3-A540D* mutants spontaneously diploidize and *mps3-Y502H* cells partially increased in ploidy at the permissive temperature of 23° (Figure 1A). This is a common phenotype in SPB duplication mutants and has been previously observed in many *mps3* mutants (Antoniacci *et al.* 2004; Jaspersen *et al.* 2002, 2006). Second, these mutants arrest in mitosis at 37° [data not shown and (Jaspersen *et al.* 2006)]. Analysis of spindle morphology in large budded mitotic cells by indirect immunofluorescence microscopy and EM revealed that two mutants, *mps3-Y502H* and *mps3-A540D*, arrested with monopolar spindles in more than 85% of cells: a single SPB associated with one array of microtubules. The *mps3-F592S* mutant arrested with either a monopolar or a short bipolar spindle (Jaspersen *et al.* 2006). The mitotic arrest and monopolar spindles are also characteristic features of SPB duplication mutants.

Two hypotheses may account for the phenotypic differences in the *mps3* SUN alleles. One possibility is that each mutant affects different aspects of SPB duplication. This idea is supported by the finding that each is suppressed to differing degrees by overexpression of the SPB components *CDC31*, *SFI1*, *MPS2*, and *NBP1* (Jaspersen *et al.* 2006). Also consistent with this idea is the recent finding that Mps3 plays a role in both SPB insertion and well as in initiation of SPB duplication (Friederichs *et al.* 2011; Jaspersen *et al.* 2002, 2006; Nishikawa *et al.* 2003). An alternative possibility is that the SUN domain may be required for additional functions of Mps3 beyond its role in SPB duplication. Overproduction of certain SPB components may or may not suppress these additional defects, such as telomere positioning/elongation, gene regulation, sister chromatid cohesion and nuclear architecture (Antoniacci *et al.* 2004, 2007; Bupp *et al.* 2007; Chan *et al.* 2011; Friederichs *et al.* 2011; Ghosh *et al.* 2012; Horigome *et al.* 2011; Oza *et al.* 2009; Schober *et al.* 2009; Witkin *et al.* 2010).

### Genome-wide screen with *mps3-SUN* alleles

**Figure 2:**
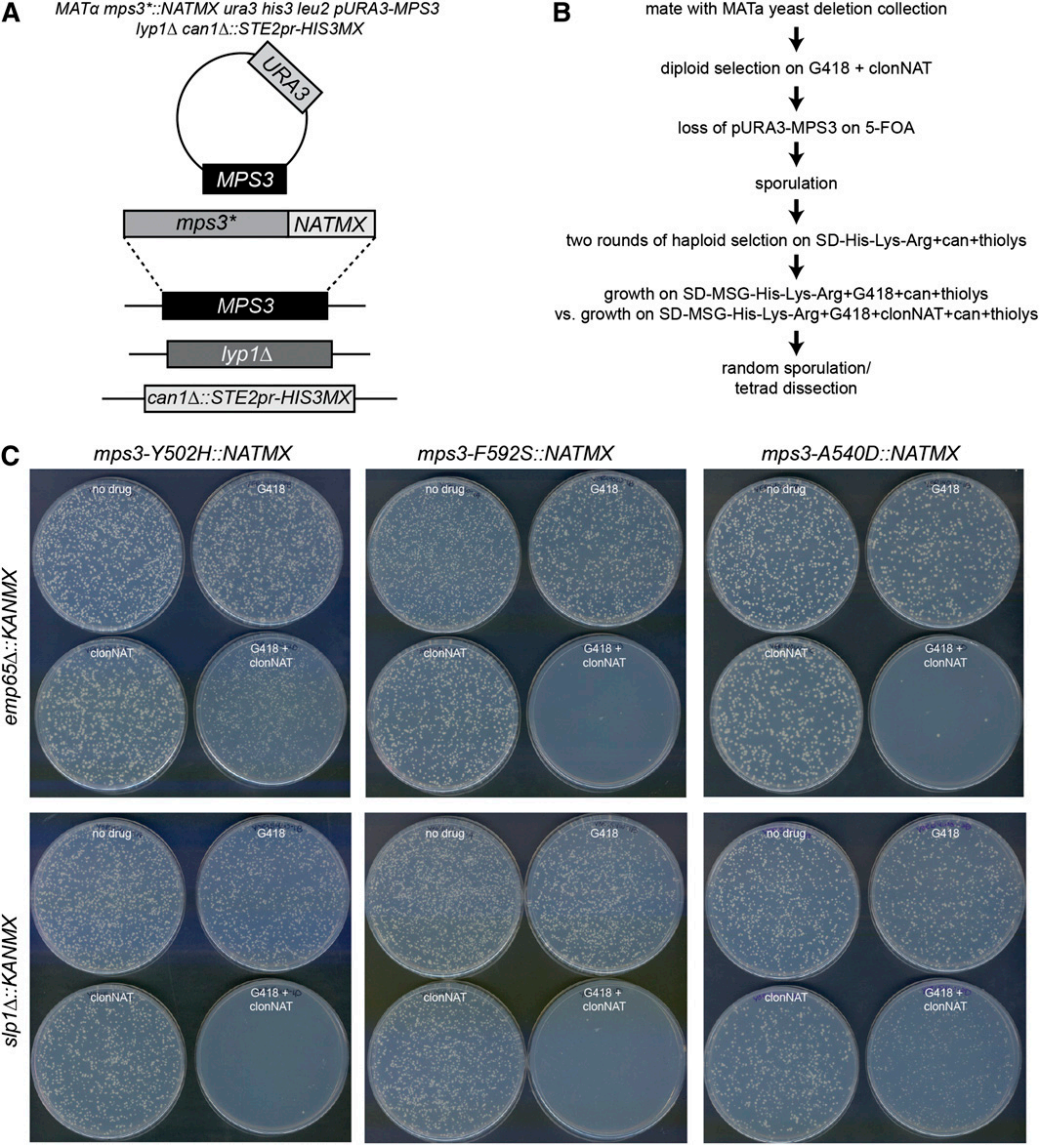
SGA analysis of *mps3* SUN domain mutants. (A) Schematic of SGA query strain. Genes encoding the lysine permease *LYP1* and the arginine permease *CAN1* are deleted in the SGA query strain. This strain also contains *S. pombe his5+* (*HIS3MX*) expressed from the *MAT****a***-specific *STE2* promoter. After addition of a *URA3*-based covering plasmid containing a wild-type copy of *MPS3*, *mps3* SUN domain mutants were integrated into the *MPS3* locus together with the *NATMX* marker. (B) Outline of SGA screening strategy used for *mps3* mutant analysis. Query strains were mated to the *MAT****a*** version of the yeast deletion collection, and diploids were selected on YPD+G418+clonNAT. *pURA3-MPS3* was removed from the diploids by growth on 5-FOA, then diploids were sporulated. Haploids were selected by growth on SD-His-Lys-Arg+thialysine+canavanine, then growth of haploids on G418 *vs.* G418+clonNAT was scored by visual inspection of the plates. Potential hits were further verified by random sporulation and/or tetrad analysis. (C) Random sporulation of *mps3-SUN* domain mutants with *emp65Δ*::*KANMX* and *slp1Δ*::*KANMX*. Sporulated cells were plated to SD-His-Lys-Arg+thialysine+canavanine (top left), SD/MSG-His-Lys-Arg+thialysine+canavanine+G418 (top right), SD/MSG-His-Lys-Arg+thialysine+canavanine+clonNAT (bottom left), and SD/MSG-His-Lys-Arg+thialysine+canavanine+G418+clonNAT (bottom right), and plates were grown for 4 d at 23°.

To better understand the role of the SUN domain in mitotic processes, we introduced *mps3-F592S*, *mps3-Y502H*, and *mps3-A540D* mutants into a query strain for synthetic genetic array analysis (SGA). Because some of these alleles result in spontaneous diploidization, the query strain contained a wild-type copy of *MPS3* on a *URA3*-marked plasmid (Figure 2A). We reconfirmed that these mutations confer a ts growth phenotype and exhibit the same changes in ploidy and mitotic arrest when the covering plasmid is removed using 5-FOA in the SGA strain background (Figure 1A). Each mutant was then mated to the collection of nonessential yeast deletions, the covering plasmid was removed after diploid selection, and sporulation was induced by growth on media lacking nitrogen. Following two rounds of haploid selection, growth of viable *MAT***a** haploids was compared at the permissive temperature of 23° on media containing G418, which selects for the deletion mutant, and media containing G418 and clonNAT, which selects for both the deletion mutant and the *mps3* SUN domain mutant allele (Figure 2B). Each screen was repeated at least three times, and a list of ∼400 gene deletions that resulted in decreased growth when combined with one or more *mps3* mutant was generated by visual inspection of plates. These 400 deletion mutants were put into a mini-array and were rescreened with each *mps3* allele two to three times, resulting in potential hits that were then confirmed by random sporulation and/or tetrad dissection. Examples of the random sporulation plates from two hits are show in Figure 2C. We performed a separate SGA screen with *mps3Δ75-150*. In total, 106 interactions were identified. Genes that we identified as inviable (synthetic lethality; SL) or slow growing (synthetically sick; SS or weakly synthetically sick; WSS) when combined with *mps3-Y502H*, *mps3-A540D*, *mps3-F592S*, or *mps3Δ75-150* are listed in Table S1.

### *mps3*-SUN alleles interact genetically with mutants in a broad range of nuclear functions

**Figure 3:**
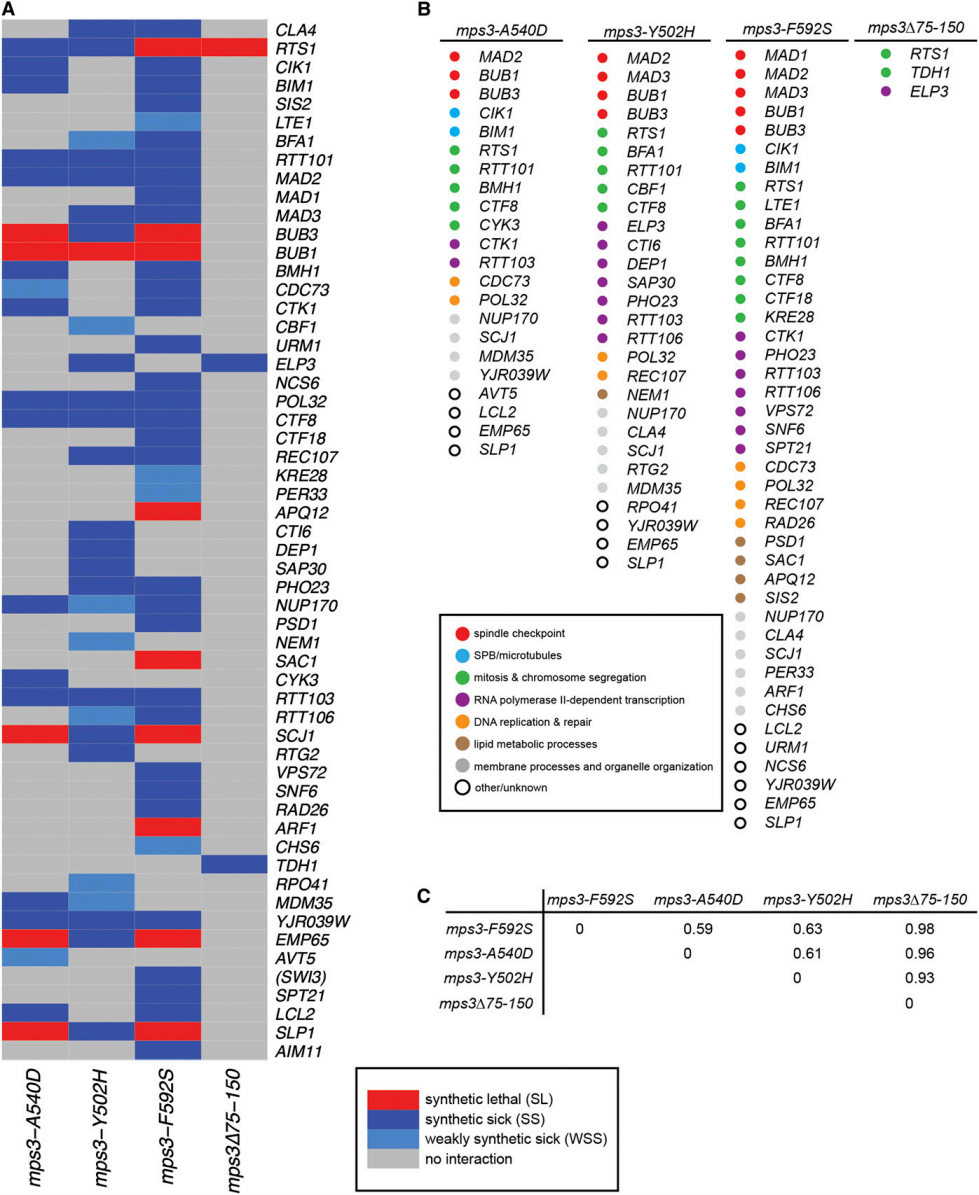
Analysis of *mps3* SUN domain interactors. (A) Two-dimensional hierarchical cluster analysis of mutants identified in *mps3* SUN domain and *mps3Δ75-150* SGA screens. Red represents synthetic lethal (SL) interactions, dark blue represents synthetic sick (SS) interactions and light blue is for weakly synthetic sick (WSS) interactions. (B) Genes identified in *mps3-A540D*, *mps3-Y502H*, *mps3-F592S*, and *mps3Δ75-150* screens are color-coded based on their cellular roles. (C) Jaccard distance between datasets is shown.

Two-dimensional hierarchical clustering shows the functional organization of hits identified for all four mutants we screened (Figure 3A). From this analysis, it is clear that *mps3-A540D*, *mps3-Y502H*, and *mps3-F592S* result in similar genetic signatures, whereas *mps3Δ75-150* has virtually no similarity with any of the SUN mutants. A comparison of datasets using Jaccard distance, which takes into account the intersection and the union of the datasets (Jay *et al.* 2012), showed an index of 0.61 for *mps3-A540D* and *mps3-Y502H* mutants, 0.59 for *mps3-F592S* and *mps3-A540D*, and 0.63 for *mps3-F592S* and *mps3-Y502H*. In contrast, an index of 0.93-0.98 was observed between *mps3Δ75-150* an each of the *mps3* SUN mutant alleles (Figure 3C). Therefore, it seems unlikely that the SUN domain mutants result in identical defects in chromosome organization and nuclear architecture as observed in *mps3Δ75-150* mutants (Bupp *et al.* 2007; Chan *et al.* 2011; Hiraga *et al.* 2012; Oza *et al.* 2009; Oza and Peterson 2010; Schober *et al.* 2009).

Does this mean that mutation of the Mps3 SUN domain does not affect nuclear organization? Given the small number of hits observed in our *mps3Δ75-150* screen, the overlap between it and the SUN mutants may have been missed. Based on the fact that all three SUN mutants interact with genes involved in chromatin structure or modification (*ELP3*, *SAP30*, *RTT106*, *PHO23*, *VPS72*, *SNF6*, *SWI3*, *INO80*), DNA replication, repair or recombination (*POL32*, *CTF8*, *RAD26*, *REC107*), and transcription/translation (*CDC73*, *CTK1*, *RTT103*, *CTI6*, *NCS6*, *NCS2*) suggests that changes in genes affecting nuclear processes exacerbates the growth defect associated with a mutation in the Mps3 SUN domain (Figure 3B, Table S1). This extensive set of genetic interactions lends considerable evidence to the idea that the *mps3* SUN mutants are at least partially defective in aspects of nuclear structure or function. However, because these interacting genes do not fall into a single obvious class of chromatin modifying enzymes or transcriptional activators/repressors, it is difficult to determine exactly what nuclear processes are affected in the absence of Mps3 SUN domain function. It also is possible that the effects of at least some of these deletions are indirect.

### *mps3*-SUN alleles interact genetically with spindle factors and spindle checkpoint components

All three SUN domain mutants also displayed growth defects when combined with deletions in genes encoding the spindle checkpoint proteins Bub1, Bub3, or Mad2, and *mps3-Y502H* and *mps3-F592S* are synthetically sick when combined with *mad3Δ* and *mps3-F592S* is synthetically sick together with *mad1Δ* [Figure 3, A and B, Table S1 (Winey and Bloom 2012)]. Genetic interactions between *mps3Δ75-150* and each of the spindle checkpoint genes were not identified, and direct testing showed that *mps3Δ75-150* is not SL or SS with mutants in any of these checkpoint factors (data not shown). These findings are consistent with the idea that the SUN domain, but not the N-terminal acidic domain, is important for Mps3′s function at the SPB (Bupp *et al.* 2007; Jaspersen *et al.* 2006). Somewhat surprisingly, only a few other genes that encode proteins affecting microtubule function or mitotic chromosome segregation were identified as SL or SS with *mps3* alleles (Figure 3, A and B, Table S1). One possible explanation for this phenomenon could be that many genes involved in mitotic spindle assembly, such as those encoding 17 of 18 SPB components, most kinetochore proteins and α-, β-, and γ-tubulin (*TUB1*, *TUB2*, and *TUB4*, respectively) are essential and are not present in the nonessential yeast deletion collection (Jaspersen and Winey 2004; Winey and Bloom 2012; Winzeler *et al.* 1999).

**Figure 4:**
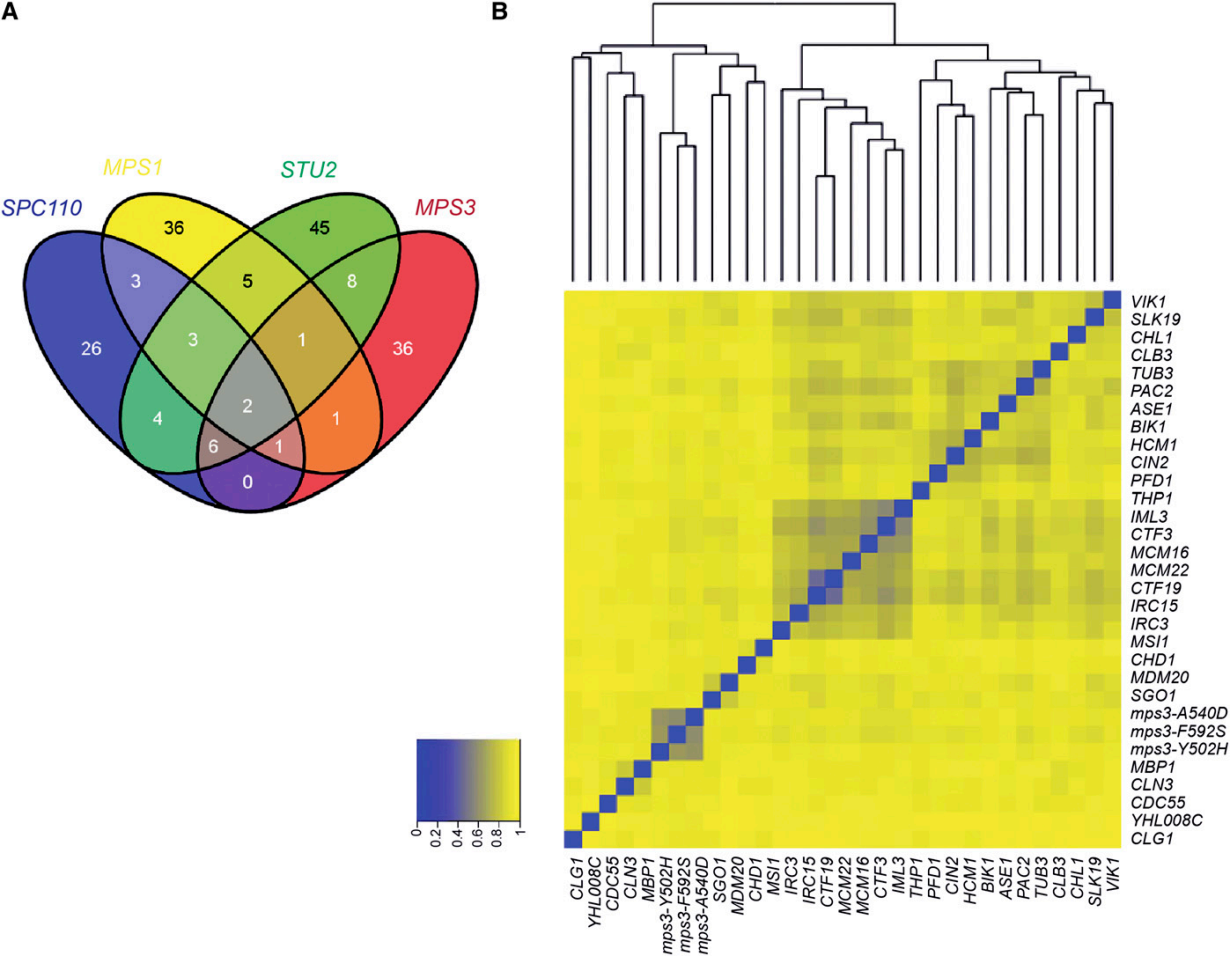
Similarity of *mps3 SUN* domain mutants to other cell cycle regulators. (A) Venn diagram showing overlap of synthetic lethal, synthetic sick or negative genetic interactions identified in analyses of *spc110*, *mps1*, *stu2*, and *mps3-SUN* domain mutants. (B) Synthetic lethal, synthetic sick, or negative genetic interactions were extracted for yeast gene pairs from BioGRID 3.1.88 and integrated with our data. To characterize the similarity of interactions found with *mps3-SUN* domain mutants to interactions identified with other deletions, we used the Jaccard distance, which takes into account the intersection and union of datasets (Jay *et al.* 2012). A total of 32 genes have a Jaccard distance <0.95 to all three *mps3-SUN* mutants, indicating a similar genetic signature. Genes were organized using two-dimensional hierarchical clustering based on their Jaccard distance from one another. Blue, Jaccard index = 0, which is very similar; yellow, Jaccard index = 1, less similar.

Comparison of our genes that are SL or SS with *mps3* SUN domain mutants to genes identified in genome-wide SGA screen for interactors of the SPB components Spc110 and Cnm67 or the SPB regulators Stu2 and Mps1 also showed only limited overlap [Figure 4A and data not shown (Costanzo *et al.* 2010; Greenland *et al.* 2010)]. Although spindle checkpoint components were required for growth of *mps1*, *mps3*, *stu2*, and *spc110* mutants, only *CTF8*, which encodes a protein involved in sister chromatid cohesion, genetically interacts with all five SPB mutants (Mayer *et al.* 2001). The limited degree of overlap indicates that *mps3* mutants affect different aspects of SPB assembly or function than the *spc110*, *cnm67*, *stu2*, or *mps1* mutants used in these studies.

Combining our genetic interaction data with that curated and annotated in BioGRID 3.1.88 as “synthetic lethal,” “synthetic growth defect,” or “negative genetic interaction,” we were able to identify genes that when mutated or deleted gave similar hits to those recovered in our *mps3* SUN domain screens. A total of 32 genes overlapped with all three *mps3* SUN mutants based on a Jaccard distance score of less than 0.95. The Jaccard distance between each pairwise combination of these genes was calculated so they could be organized by two-dimensional hierarchical clustering, as shown in Figure 4B. Included in this list were kinetochore components (*CTF3*, *MCM16*, *MCM22*, *CTF19*, *SLK19*), the minor α-tubulin (*TUB3*), and proteins involved in tubulin folding (*CIN2*, *PFD1*, *PAC2*), microtubule-associated factors (*VIK1*, *ASE1*, *BIK1*, *IML3*, *IRC15*), and cell-cycle regulators (*SGO1*, *MBP1*, *CLN3*, *CLB3*, *CDC55*, *CLG1*). The fact that *mps3* SUN domain mutants have genetic interactions similar to other cell cycle regulators and factors involved in mitotic spindle assembly is consistent with the idea that the *mps3* SUN domain mutants have a defect in SPB duplication. Genes encoding the chromatin modifiers *MSI1* or *CHD1*, the N-terminal acetyltransferase *MDM20*, the TREX complex subunit *THP1*, the sister chromatid cohesion factor *CHL1* and the forkhead transcription factor *HCM1* were also identified in this analysis, suggesting that the SUN domain is required for Mps3′s function in nuclear organization.

### *SLP1* and *EMP65* encode conserved integral membrane proteins that are essential for growth of *mps3*-SUN domain mutants

In addition to genes that affect spindle function or nuclear organization, our *mps3* SUN SGA screens also were enriched in genes affecting additional biological processes that may shed light into novel functions of SUN proteins during cell division. We found that a number of genes implicated in mitochondrial function (*MDM35*, *RPO41*, *RTG2*, *MRP4*, *YJR039W*), lipid biosynthesis (*DEP1*, *NEM1*, *SAC1*, *PSD1*, *SIS1*, *APQ12*), and other aspects of organelle biogenesis and transport (*NUP170*, *PER33*, *CHS6*, *BMH1*, *PMA1*, *AVT5*; Figure 3B, Table S1) displayed genetic interactions with all three *mps3* SUN domain mutants. Three poorly characterized ORFs suspected to play a role in ER function based on genetic interactions in high-throughput screens also were identified in our screens (Jonikas *et al.* 2009). These factors (*LCL2*, *SLP1*, *EMP65/YER140W*) are ideal candidates to participate in folding or targeting of Mps3 or in other functions needed for SPB duplication because integral membrane proteins needed for SPB assembly must be first inserted into the ER and then transported to the NE (Burns and Wente 2012; Jaspersen and Ghosh 2012). Because *emp65Δ* and *slp1Δ* were SS or SL with all three *mps3* SUN domain mutants (Figure 2C, Figure 3, Table S1), we focused on further characterizing the function of Emp65 and Slp1.

**Figure 5:**
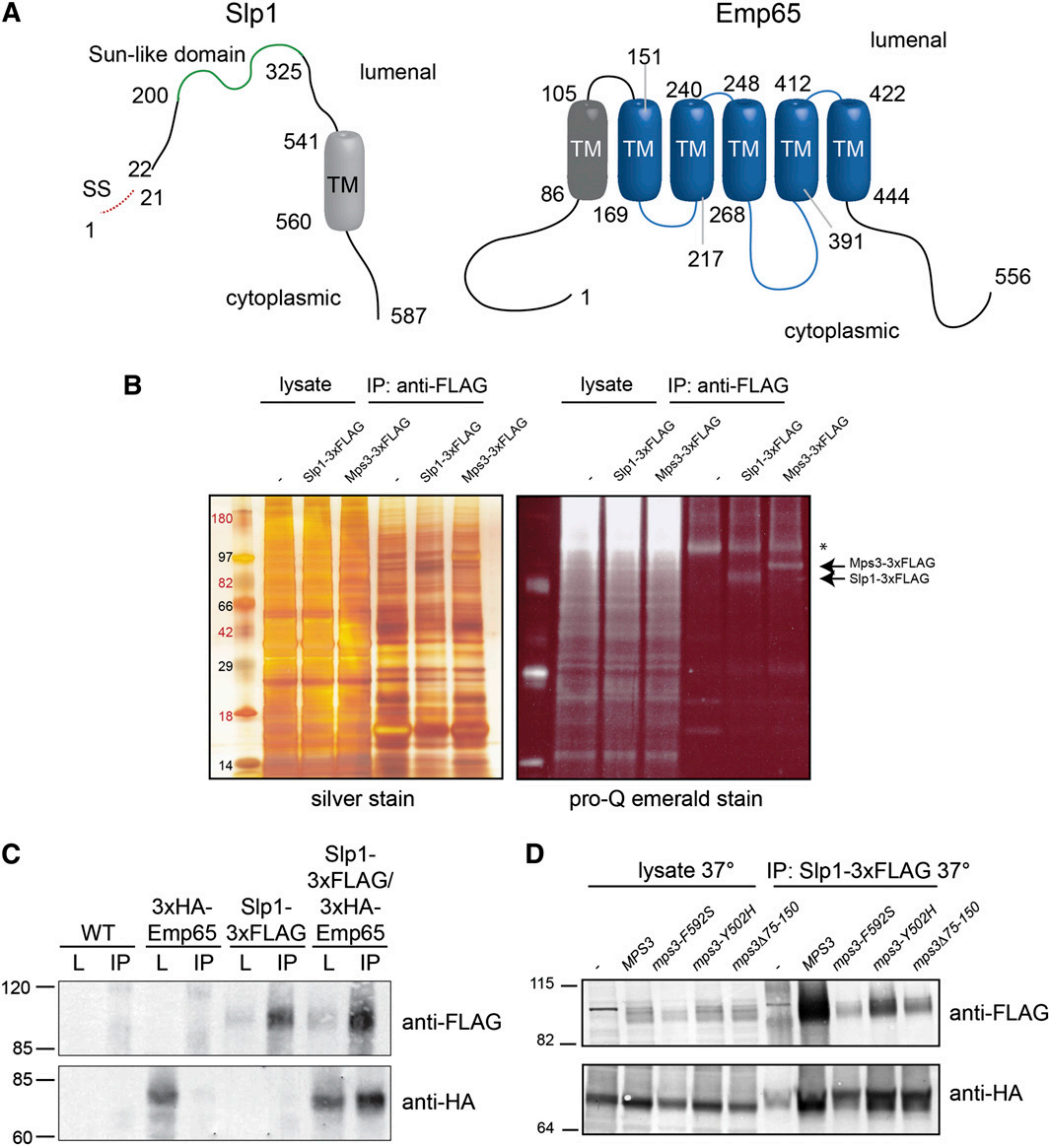
Slp1 and Emp65 form an integral membrane protein complex. (A) Schematic of Slp1 and Emp65. Slp1 contains a putative signal sequence from amino acids 1-21, a SUN-like domain from residues 200-325 and a predicted transmembrane segment from residues 541-560. Emp65 contains 6 predicted transmembrane segments from residues 86−105, 151−169, 217−240, 248−268, 391−412, and 422−444. The predicted topology of both proteins is shown (Bernsel *et al.* 2009; Reynolds *et al.* 2008). (B) Lysates from wild-type (SLJ001), Mps3-3xFLAG (SLJ3529), and Slp1-3xFLAG (SLJ3864) cells were prepared by cryolysis, and proteins were immunoprecipitated using anti-FLAG-M2 beads and separated by SDS-PAGE. Proteins in lysates and immunoprecipitates were visualized by silver staining or by Pro-Q Emerald staining, which detects glycosylated proteins. (C) Wild-type (SLJ001), *SLP1-3xFLAG* (SLJ3863), *GAL-3xHA-EMP65* (SLJ3837), and *SLP1-3xFLAG GAL-3xHA-EMP65* (SLJ4048) were grown to log phase in YPGR, harvested, lysed by liquid nitrogen grinding, and lysates were used in immunoprecipitation assays with anti-FLAG-M2 beads. Both the lysate and the immunoprecipitates were analyzed by SDS-PAGE followed by western blotting with anti-HA and anti-FLAG antibodies. (D) *GAL-3xHA-EMP65* (SLJ6064), *SLP1-3xFLAG GAL-3xHA-EMP65* (SLJ6088), *mps3-F592S SLP1-3xFLAG GAL-3xHA-EMP65* (SLJ6086), *mps3-Y502H SLP1-3xFLAG GAL-3xHA-EMP65* (SLJ6087), *mps3Δ75-150 SLP1-3xFLAG GAL-3xHA-EMP65* (SLJ6092) cells grown in YPGR at 23° were shifted to 37° for 3 hr, harvested, lysed by liquid nitrogen grinding, and lysates were used in immunoprecipitation assays with anti-HA beads. Both the lysate and the immunoprecipitates were analyzed by SDS-PAGE followed by western blotting with anti-HA and anti-FLAG antibodies. (B−D) Positions of molecular weight markers are indicated on the left.

*EMP65* and *SLP1* are predicted to encode integral membrane proteins. The 587-amino acid *S. cerevisiae* Slp1 protein contains a signal sequence from residues 1−21, a transmembrane domain from residues 541−560, and a central domain from residues 200−325 that bears resemblance to the all β-fold domain found in Mps3 and other SUN proteins (Figure 5A). This SUN-like domain is found in virtually all eukaryotes (Jaspersen *et al.* 2006), but unlike its SUN domain counterparts that are typically located at or near the C-terminus of the protein, the SUN-like domain most often is located in the central region of the protein. Topology prediction tools indicate that the SUN-like region is most likely located in the lumenal space (Bernsel *et al.* 2009; Reynolds *et al.* 2008). *EMP65* is predicted to encode a 556-amino acid protein containing at least six transmembrane domains (Figure 5A). Residues 147−453 are part of a conserved domain of unknown function (PFAM DUF747) present in virtually all eukaryotes. This domain was originally thought to be a human cytomegalovirus receptor, but more recently it was identified in a mouse transmembrane protein required for normal skeletal development (Baldwin *et al.* 2000; Howell *et al.* 2007). The most likely orientation of Emp65 is with its N and C-termini facing the cytoplasm, as shown in Figure 5A (Bernsel *et al.* 2009; Reynolds *et al.* 2008).

To identify genes involved in the unfolded protein response, Jonikas *et al.* screened the yeast deletion collection for mutants that failed to induce an unfolded protein response element reporter construct in the presence of the reducing agent dithiothreitol (DTT). They identified both *EMP65* and *SLP1* in this study (Jonikas *et al.* 2009). The similar response of both deletions to unfolded proteins and pattern of genetic interactions suggested that the proteins encoded by *EMP65* and *SLP1* most likely form a complex *in vivo* and function in folding of integral membrane proteins. Emp65 also was identified as a putative mitochondrial protein because it was significantly enriched in highly purified mitochondrial fractions in high throughput studies (Reinders *et al.* 2006; Sickmann *et al.* 2003). Slp1 was not found in these preparations and functional data linking Emp65 to the mitochondria has not been discovered, so the significance of this finding is currently unclear. Given the fact that Slp1 and Emp65 are highly conserved and appear to act together in a complex, we were interested in understanding their function, including why they are required for viability of *mps3* SUN domain mutants.

### Slp1 and Emp65 form an ER membrane-associated complex

As a first step toward achieving this goal, we wanted to examine the distribution of both proteins. We fused *SLP1* to three copies of the FLAG epitope using PCR-based methods and tested that *SLP1-3xFLAG* encoded a fully functional fusion protein by verifying it was able to rescue the growth defect of *mps3-F592S* cells (data not shown). Slp1-3xFLAG is predicted to migrate at approximately 70 kDa based on its amino acid composition; however, we observed a series of heterogeneous bands migrating at approximately 100 kDa on SDS-PAGE (Figure 5, B−D). This is most likely due to glycosylation because immunoprecipitated Slp1-3xFLAG can be visualized by Pro-Q Emerald stain, which detects periodate-oxidized carbohydrates (Figure 5B). The Slp1 coding sequence contains six predicted N-linked glycosylation sites: N24, N377, N380, N407, N447, and N485, as well as several possible O-linked glycosylation sites. The fact that Slp1-3xFLAG is glycosylated strongly suggests it is an integral membrane protein, similar to Mps3-3xFLAG, which is also glycosylated [Figure 5B) Nishikawa *et al.* 2003)]. We verified that Slp1 was an integral membrane protein by testing the ability of different reagents to solubilize Slp1-3xFLAG from the pellet fraction of yeast spheroplasts. Like Mps3 (Jaspersen *et al.* 2002), Slp1-3xFLAG was only extracted from the pellet in the presence of the detergent Triton X-100, confirming it is an integral membrane protein (data not shown).

Despite repeated attempts, we were unable to detect the expression of a variety of endogenously tagged versions of Emp65 when using western blotting (data not shown). Therefore, we created N-terminally tagged versions of *EMP65* under the control of the galactose-inducible *GAL1* promoter as the sole copy of *EMP65* in the cell. Growth of these cells on galactose did not result in any obvious growth defect on YPGR (Figure S2). In addition, cells containing N-terminally tagged versions of *EMP65* were able to grow in the presence of all three ts mutants in the Mps3 SUN domain, indicating that epitope tagged versions of Emp65 are functional (Figure S2 and data not shown). *mps3-A540D GAL-GFP-EMP65* or *mps3-F592S GAL-GFP-EMP65* are most likely viable on glucose-containing media as the result of leaky expression of *EMP65* from the *GAL1* promoter. As was the case for Slp1, 3xHA-Emp65 appeared as a heterogeneous band on SDS-PAGE (Figure 5, C and D). It too cofractionated with membranes and was only partially extractable with detergent and salt, indicating that it is also an integral membrane protein (data not shown). However, we were not able to detect it using Pro-Q Emerald stain (data not shown). Examination of Emp65 amino acid sequence showed that it contains a single putative site of glycosylation at N317. It is possible that this single site is below the detection limit for the stain, or alternatively, Emp65 may not be a glycoprotein.

To test whether Slp1 and Emp65 form a membrane-associated protein complex, we prepared extracts from cells containing 3xHA-Emp65 and/or Slp1-3xFLAG by cryolysis. Slp1-3xFLAG was immunoprecipitated using anti-FLAG resin, and copurifying proteins were analyzed by western blotting with both anti-HA and anti-FLAG antibodies. 3xHA-Emp65 coimmunoprecipitated with Slp1-3xFLAG (Figure 5C). The fact that 3xHA-Emp65 did not coprecipitate in cells lacking Slp1-3xFLAG and that we could also perform the coimmunoprecipitation using anti-HA beads and recover Slp1-3xFLAG in the 3xHA-Emp65 complex suggests that binding is specific (Figure 5D and data not shown). Based on these data, we conclude that Slp1 and Emp65 form a membrane-associated protein complex.

**Figure 6:**
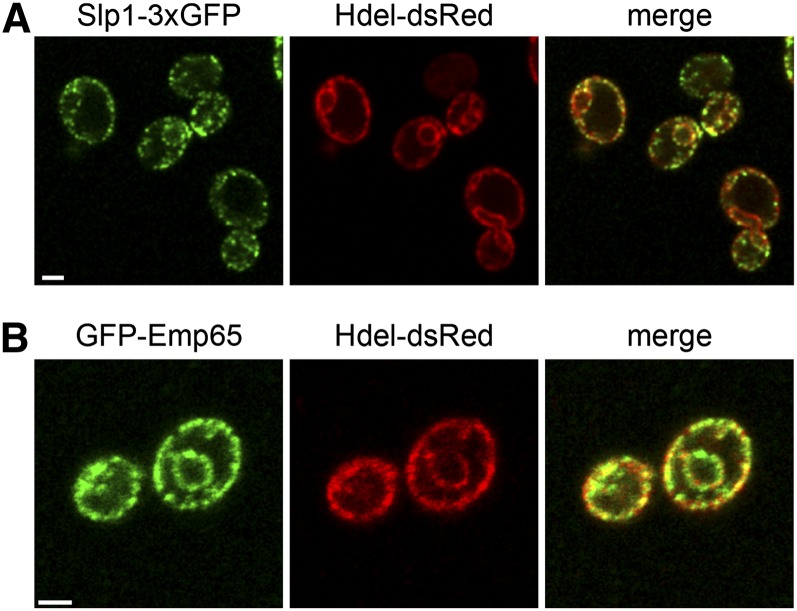
Slp1 and Emp65 are ER-membrane proteins. Cells containing Slp1-3xGFP (A, green; SLJ6525) or GFP-Emp65 (B, green; SLJ4074), and the ER-marker HDEL-dsRed (red) were visualized by confocal imaging. Bars, 2 µm.

To examine the subcellular distribution of Slp1 and Emp65, we fused the endogenous copy of *SLP1* to three copies of GFP (Slp1-3xGFP) or replaced *EMP65* with an overexpressed version tagged with GFP at the N-terminus (GFP-Emp65). As shown in Figure 6, both Slp1-3xGFP and GFP-Emp65 appeared in a punctate pattern around the cell periphery and around the nucleus. This staining pattern is reminiscent of the ER membrane, which is adjacent to the NE and cell cortex in budding yeast. Colocalization with the lumenal ER marker HDEL-dsRed showed significant overlap (Figure 6). Slp1-3xGFP did not co-localize with the plasma membrane proteins Pil1 or Pma1, with the NE protein Nup49 or with mitochondria or endocytic vesicles, which were visualized using the vital dyes mitotracker red and FM4-64, respectively (data not shown). Therefore, we conclude that Slp1 and Emp65 form a novel ER-membrane associated complex.

### Slp1 and Emp65 complex formation is Mps3-independent

**Figure 7:**
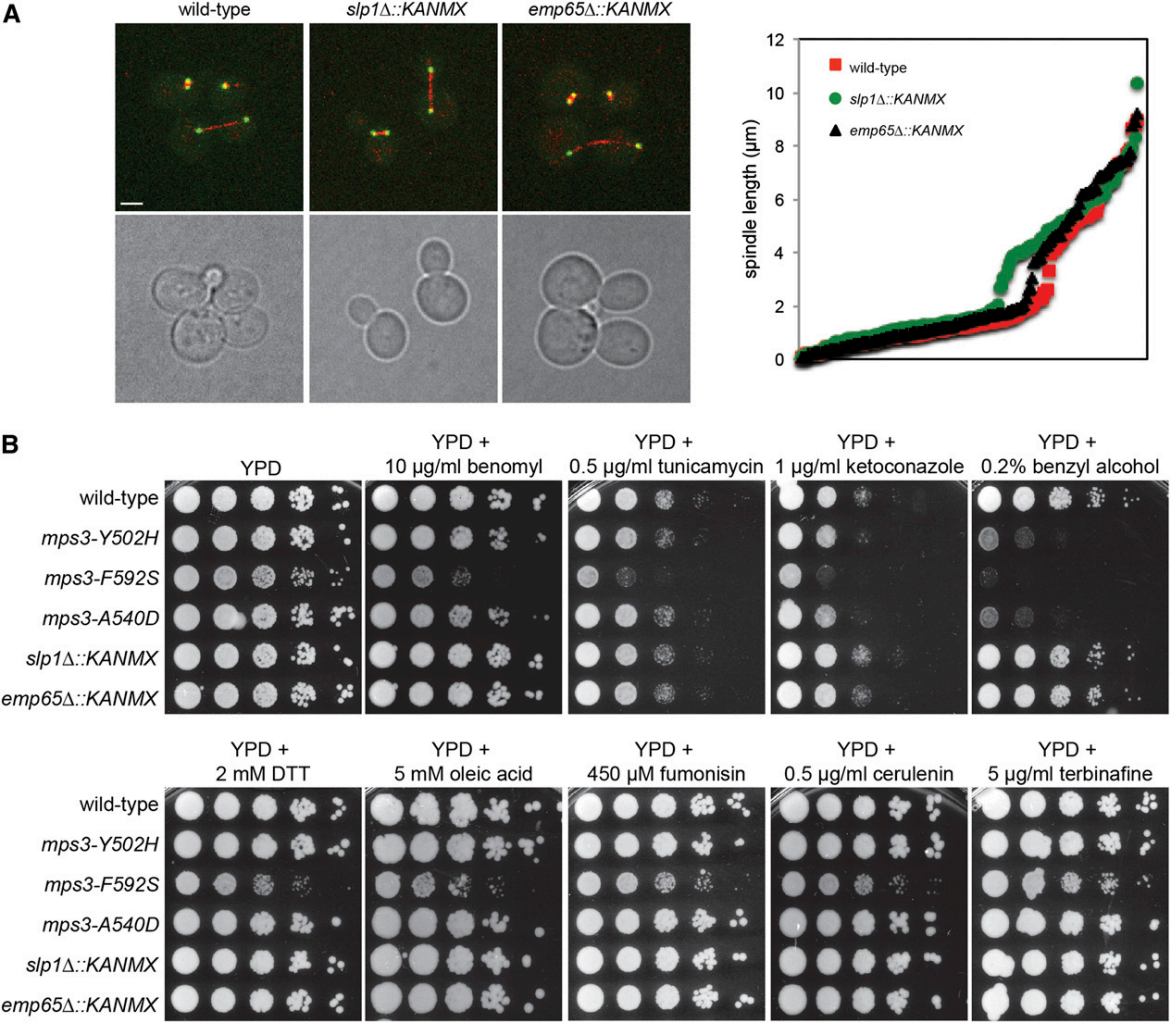
Mitotic spindle formation is Slp1 and Emp65-independent. (A) Wild-type (SLJ6430), *slp1Δ* (SLJ6434), and *emp65Δ* (SLJ6436) cells containing *TUB4-GFP* and *mCherry-TUB1* to visualize SPBs (green) and microtubules (red), respectively, were grown to mid-log phase in YPD at 30° and imaged by confocal microscopy. The length of the mitotic spindle in large budded cells was measured using the distance between the two SPBs. A plot of these distances for 200 mitotic nuclei from each cell type is shown. (B) Wild-type (SLJ771), *slp1Δ* (SLJ3136), *emp65Δ* (SLJ3277), *mps3-A540D* (SLJ1622), *mps3-Y502H* (SLJ1712), and *mps3-F592S* (SLJ1711) cells were serially diluted 10-fold and spotted onto YPD, YPD+10 µg/mL benomyl, YPD+0.5 µg/mL tunicamycin, YPD+1 µg/mL ketoconazole, YPD+0.2% benzyl alcohol, YPD+2 mM DTT, YPD+5 mM oleic acid, YPD+450 µM fumonisin, YPD+0.5 µg/mL cerulenin, and YPD+5 µg/mL terbinafine. Cells were grown for 2−4 d at 23°.

The fact that both Slp1 and Mps3 contain related domains and that *slp1Δ* or *emp65Δ* are synthetically lethal with *mps3* SUN domain mutants defective in SPB duplication raise the possibility that the Slp1-Emp65 complex plays a partially redundant function(s) with Mps3 *in vivo*. Several lines of evidence suggest that Slp1 and Emp65 do not function in SPB duplication, however. First, neither Slp1-3xGFP nor GFP-Emp65 localize to the SPB (Figure 6 and data not shown). Second, analysis of *slp1Δ* or *emp65Δ* mutants did not reveal any defects in microtubule function, including mitotic spindle assembly or spindle positioning (Figure 7A). Analysis of spindle length in large-budded wild-type, *slp1Δ*, and *emp65Δ* cells showed that both deletions do not appear to affect the average length of the mitotic spindle in metaphase or anaphase compared to wild-type controls: metaphase spindles were 0.84 ± 0.39 µm (n = 111), 0.91 ± 0.40 µm (n = 83, *P* = 0.22) and 0.78 ± 0.39 µm (n = 102, *P* = 0.26) whereas anaphase spindles were 4.6 ± 2.2 µm (n = 64), 4.7 ± 1.3 µm (n = 83, *P* = 0.81), and 4.5 ± 2.3 µm (n = 63, *P* = 0.74) in wild-type, *slp1Δ* and *emp65Δ* cells, respectively (Figure 7A, *P* value compared with wild-type using Student’s *t*-test). In addition, *slp1Δ* or *emp65Δ* mutants do not exhibit any obvious cell-cycle delay or show increased sensitivity to the microtubule depolymerizing drug benomyl (Figure 7B), which are common phenotypes of SPB mutants including our *mps3* SUN domain mutants (Antoniacci *et al.* 2004; Jaspersen *et al.* 2002, 2006; Nishikawa *et al.* 2003). Lastly, *slp1Δ* and *emp65Δ* are not lethal in combination with other SPB duplication mutants, including those involved in half-bridge assembly and elongation, satellite formation, microtubule nucleation and SPB insertion into the NE [Table 1 (Jaspersen and Winey 2004; Winey and Bloom 2012)].

**Table 1:** Genetic interactions between *slp1Δ or emp65Δ* and SPB mutants

SPB Mutant	SPB Defect	*slp1Δ*::*KANMX*	*emp65Δ*::*KANMX*
*mps3Δ75-150*	None	−	−
*mps3-Y502H*	HB assembly	SL	SS
*mps3-A540D*	HB assembly	SS	SL
*mps3-F592S*	HB assembly, SPB insertion	SL	SL
*mps3-1*	HB assembly	−	−
*cdc31-2*	HB assembly	−	−
*kar1Δ17*	HB assembly	−	−
*sfi1-3*	HB assembly	−	−
*sfi1-7*	HB elongation	−	−
*spc42-11*	satellite formation	−	−
*spc29-3*	satellite formation	−	−
*spc110-220*	nMT assembly	−	−
*tub1-4*	nMT and cMT assembly	−	−
*bbp1-1*	SPB insertion	−	−
*ndc1-39*	SPB insertion	−	−
*mps2-1*	SPB insertion	−	−
*mps1-8*	satellite formation	−	−

All genetic interactions were scored on YPD at 23°. SL, synthetic lethal; SS, synthetic sick; -, no genetic interaction.

Therefore, it seems most probable that Slp1 and Emp65 only indirectly affect Mps3 function. Consistent with this idea, we found that Mps3 does not associate with Slp1-Emp65 *in vivo*; immunoprecipitation of Mps3-3xHA or Mps3-3xFLAG failed to pull-down Slp1-3xFLAG or 3xHA-Emp65, respectively (Figure S3, A and B). In addition, binding of 3xHA-Emp65 with Slp1-3xFLAG was not affected by loss of Mps3 function because the proteins still coimmunoprecipitated from lysates prepared from wild-type, *mps3-F529S*, *mps3-Y502H*, *mps3-A540D*, and *mps3Δ75-150* mutants grown at the nonpermissive temperature of 37° (Figure 5D). Thus, the ability of Slp1 and Emp65 to form a complex does not appear to require Mps3 function.

### Slp1 is required for localization of Mps3 to the NE

**Figure 8:**
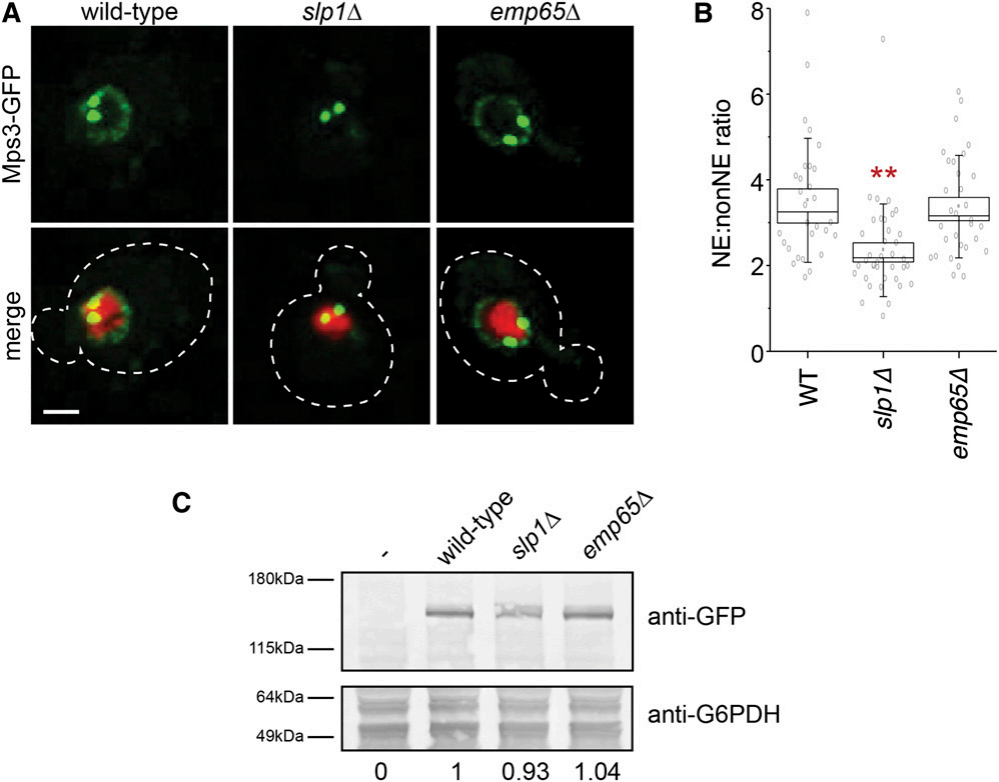
Slp1 and Emp65 are required for Mps3 localization to the NE. (A) Localization of Mps3-GFP (green) and H2B-mCherry (red) in wild-type (SLJ5669), *slp1Δ* (SLJ6783), or *emp65Δ* (SLJ6785). The cell is outlined in white based on the DIC image. Bar, 2 µm. (B) Quantitation of NE/non-NE ratio in Mps3-GFP in images from (A) was performed as previously described (Gardner *et al.* 2011). Values for each data point (gray circles), the mean (black square) and median values (line), SE (box) and SD (lines) for each sample are depicted. Values that are statistically significant from wild-type (*P* < 10^−4^) are indicated with an asterisk. (C) Protein levels in whole-cell extracts of cells from (A) were determined by western blotting with anti-GFP antibodies. Glucose-6-phosphate dehydrogenase (G6PDH) serves as a loading control and allows for normalization of the levels of Mps3-GFP in different strains (below). The strain lacking Mps3-GFP was assigned a value of 0 whereas the wild-type strain containing Mps3-GFP was given a value of 1.

Because Slp1 and Emp65 are suspected to play a role in folding of integral membrane proteins (Jonikas *et al.* 2009), we considered the possibility that they were required for Mps3 processing, including Mps3 stability, posttranslation modification, and/or localization. Western blotting of Mps3-GFP from wild-type, *slp1Δ* and *emp65Δ* cells showed it was present at roughly equivalent levels and migrated at the same position on SDS-PAGE (Figure 8C). Therefore, *slp1Δ* and *emp65Δ* mutants do not affect Mps3 synthesis or stability. However, when we examined Mps3-GFP localization, we observed a significant decrease in the amount of Mps3-GFP at the NE *vs.* the amount present at non-NE membranes in s*lp1Δ* mutants compared with wild-type (NE/non ratio wild-type = 3.52 ± 0.0.5, *slp1Δ*=2.36 ± 0.03, *P* < 0.0001 based on Mann-Whitney test; Mps3-GFP was at the SPB was excluded from this analysis; see *Materials and Methods*; Figure 8, A and B). The level of Mps3-GFP at the NE was not affected in *emp65Δ* mutants (NE/non ratio = 3.37 ± 0.04, *P* = 0.35 compared with wild-type; Figure 8, A and B). Levels of Mps3-GFP at the SPB were equivalent in wild-type, *slp1Δ*, and *emp65Δ* cells (data not shown), consistent with our finding that *slp1Δ* and *emp65Δ* do not have defects in SPB duplication and spindle assembly. Therefore, Slp1, but not Emp65, is required for Mps3 accumulation within the NE.

Previous work suggested that deletion of *SLP1* or *EMP65* trigger the unfolded protein response (Jonikas *et al.* 2009). In our W303-based strain background, *slp1Δ* and *emp65Δ* did not display sensitivity to tunicamycin or DTT (Figure 7B), two drugs that induce the accumulation of unfolded proteins and are frequently toxic to yeast mutants unable to respond to the ER stress caused by protein misfolding. This finding raises the issue as to whether unfolded proteins, including *mps3* SUN domain mutant proteins, accumulate in *slp1Δ* and *emp65Δ* mutants. Microarray analysis of *slp1Δ* cells failed to identify a significant up-regulation of genes involved in protein folding or the unfolded protein response compared to wild-type (data not shown). While it is possible that Slp1 is required for folding of other unknown integral membrane proteins, our data for Mps3-GFP instead suggests a role in NE localization or tethering.

## DISCUSSION

In this article, we have shown that the Mps3 SUN domain is essential for mitotic growth and for sporulation. Previous work suggested strain-background specific requirements for the Mps3 SUN domain, possibly due to mutations in factors that restrict Mps3 to the SPB (Rao *et al.* 2011). Using identical alleles, we compared multiple SUN domain mutants in three commonly used *S. cerevisiae* strain backgrounds: W303, BY (a S288c derivative), and SK-1. A precise deletion of the SUN domain (amino acids 415−645) is lethal in all three, confirming the idea that the SUN domain is essential for viability. Mutation of conserved residues or deletion of the second half of the SUN domain resulted in ts alleles due to a defect in SPB duplication and chromosome segregation. The SUN domain is also critical for meiosis as evidenced by the decreased number of viable tetrads formed when *2xmps3ΔSUN2* is combined with *mps3ΔSUN*, *mps3ΔSUN1*, *mps3ΔSUN2*, or *2xmps3ΔSUN2*. Interestingly, the Mps3 N-terminal acidic domain (residues 75−150) is not required for vegetative growth or for sporulation. Although this region is required for Mps3 localization to the INM and for chromosome positioning during mitosis (Bupp *et al.* 2007; Gardner *et al.* 2011; Schober *et al.* 2009), it appears that additional portions of the N-terminus function in meiotic chromosome pairing and telomere-led chromosome movements (Conrad *et al.* 2007, 2008; Lee *et al.* 2012).

How might mutations in the SUN domain affect chromosome positioning within the nucleus? One possibility is that the Mps3 levels in these mutants are reduced and there is simply not enough Mps3 on the NE to anchor chromosomes or chromosome binding proteins on the membrane. Consistent with this idea, mps3-F592S-GFP is undetectable on the NE in mitotic cells at the permissive temperature (Gardner *et al.* 2011). Another possibility, which is not mutually exclusive, is that the linker complex connecting the INM and ONM is defective in the absence of the SUN domain. Based on studies in higher eukaryotes, there is ample evidence to support this idea, including recent data demonstrating roles for mSun1 and mSun2 in the efficient repair of DNA breaks and for mSun1 and *C. elegans* SUN-1 in meiotic chromosome dynamics (Hiraoka and Dernburg 2009; Lei *et al.* 2012; Morimoto *et al.* 2012; Penkner *et al.* 2009; Sato *et al.* 2009).

Perhaps not surprisingly, *mps3* SUN domain mutants exhibited negative genetic interactions with chromosome segregation factors and components of the spindle checkpoint. The primary defect in these mutants is an inability to duplicate the SPB and form a functional bipolar spindle (Jaspersen *et al.* 2006). Mechanistic insight as to the role of Mps3 during SPB duplication comes from the observation that deletions of lipid metabolic enzymes and factors involved in membrane organization exacerbated the growth defect of *mps3* SUN mutants, but not *mps3Δ75-150* mutants. This finding suggests that the SUN domain may play a role in modulating NE dynamics, a function that could be linked to its role at the SPB. Insertion of large protein complexes such as the NPC or SPB requires the formation of a pore membrane: a highly curved membrane site at which the INM and ONM are contiguous (Jaspersen and Ghosh 2012). Studies of NPC assembly suggest that lipid remodeling and membrane deformation by the reticulons are important in the early steps of pore membrane formation, and membrane coat proteins such as the ALPS (for ArfGAP1 lipid packing sensor) domain containing proteins bind and stabilize the curved membrane (Dawson *et al.* 2009; Hetzer and Wente 2009). Although less is known about the formation of the membrane pore at the SPB, recent work suggests that similar membrane remodeling events occur during both NPC and SPB duplication (Casey *et al.* 2012; Jaspersen and Ghosh 2012; Kupke *et al.* 2011). Analysis of a dominant allele of *MPS3* in budding yeast and two-hybrid analysis of Sad1 binding proteins in fission yeast suggest that SUN proteins may tether proteins involved in membrane organization at specific sites in the NE, such as at the site of SPB insertion (Friederichs *et al.* 2011; Miki *et al.* 2004). At least four possible candidates where identified in our screen: Nem1, a regulator of nuclear morphology and phospholipid biosynthesis (Campbell *et al.* 2006; Santos-Rosa *et al.* 2005; Siniossoglou *et al.* 1998), Apq12, a NE protein required for NPC assembly at low temperatures and for SPB insertion in *S. pombe* (Baker *et al.* 2004; Scarcelli *et al.* 2007; Tamm *et al.* 2011), Per33, a transmembrane ER and NPC-associated protein (Chadrin *et al.* 2010), and the nucleoporin Nup170, which forms part of the core scaffold essential for anchoring the NPC in the NE (Alber *et al.* 2007; Makio *et al.* 2009; Onischenko *et al.* 2009). When taken together, our genetic data point to a possible new function of SUN domain containing proteins in membrane dynamics that may account for their role at both SPBs/centrosomes and at NPCs (Liu *et al.* 2007; Talamas and Hetzer 2011).

The two most striking interactors were *slp1Δ* and *emp65Δ*, both of which strongly inhibited growth of all three *mps3* SUN domain mutants. As suggested by both our genetic interactions and those of Jonikas *et al.*, Slp1 and Emp65 form a membrane-associated protein complex (Jonikas *et al.* 2009). Two high throughput proteomics analyses suggested that Emp65, but not Slp1, was associated with mitochondria (Reinders *et al.* 2006; Sickmann *et al.* 2003). However, we were unable to observe significant colocalization of GFP-Emp65 with mitotracker red, a dye that allows visualization of mitochondria (data not shown). Rather, our data suggest that Slp1-Emp65 are present on both cortical and perinuclear ER membranes where they form a membrane-associated complex.

If Slp1 and Emp65 are part of a complex, why is Mps3 localization affected by deletion of *SLP1* but not deletion of *EMP65*? One possibility is that the Slp1-Emp65 is required for a process that indirectly affects Mps3 function. Although it is possible that they are involved in membrane protein folding as previously suggested (Jonikas *et al.* 2009), the fact that both mutants are unaffected by DTT and tunicamycin, which trigger the unfolded protein response and inhibit growth of yeast with protein folding defects, argues against this possibility. In addition, the total levels of Mps3 protein are comparable between *slp1Δ*, *emp65Δ* and wild-type, based on western blotting and total fluorescence intensity of Mps3-GFP. Therefore, we favor the possibility that Slp1 may perform additional functions, including localization of NE proteins. How Slp1 might do this is currently unknown. However, it is likely that the effects of Slp1 are indirect since we were unable to detect binding between Slp1 and Mps3. Because NE levels of the mps3 SUN domain mutant proteins are dramatically reduced compared to wild-type (Gardner *et al.* 2011; Jaspersen *et al.* 2006), we hypothesize that any additional decrease in levels of the mps3 mutant protein would result in reduced fitness or lethality. Although the NE pool of Mps3 is most likely not essential for growth, it serves as the SPB reservoir (Bupp *et al.* 2007; Gardner *et al.* 2011). Slp1 may help maintain this pool of Mps3 and promote exchange of Mps3 between the NE and SPB.

## Supplementary Material

Supporting Information
