# Supplementary material for: Genetic Analysis of Mps3 SUN Domain Mutants in Saccharomyces cerevisiae Reveals an Interaction with the SUN-Like Protein Slp1
Source: G3 (Bethesda). 2012 Dec 1;2(12):1703–18. doi: 10.1534/g3.112.004614 (PMC3516490; doi:10.1534/g3.112.004614)
Supplement: Supporting Information [file supp_2_12_1703__index.html]

Supporting Information 

# Genetic Analysis of Mps3 SUN Domain Mutants in *Saccharomyces cerevisiae* Reveals an Interaction with the SUN-Like Protein Slp1

## Supporting Information for Friederichs *et al.*, 2012

**Files in this Data Supplement:**

- Supporting Information - Figures S1-S3 and Tables S1 and S2 (PDF, 3.4 MB)
- Figure S1 - Sequence alignment of SUN domain (PDF, 542 KB)
- Figure S2 - GAL-EMP65 does not affect cell growth (PDF, 1.7 MB)
- Figure S3 - Mps3 does not co-immunoprecipitate with Slp1 or Emp65 (PDF, 1.9 MB)
- Table S2 - Yeast strains used in this study (PDF, 111 KB)
- Table S1 - Genetic interactions with *mps3* alleles (.xls, 145 KB)
