# Supplementary material for: Genetic Analysis of Mps3 SUN Domain Mutants in Saccharomyces cerevisiae Reveals an Interaction with the SUN-Like Protein Slp1
Source: G3 (Bethesda). 2012 Dec 1;2(12):1703–18. doi: 10.1534/g3.112.004614 (PMC3516490; doi:10.1534/g3.112.004614)
Supplement: Supporting Information [file supp_2.12.1703_004614SI.pdf]

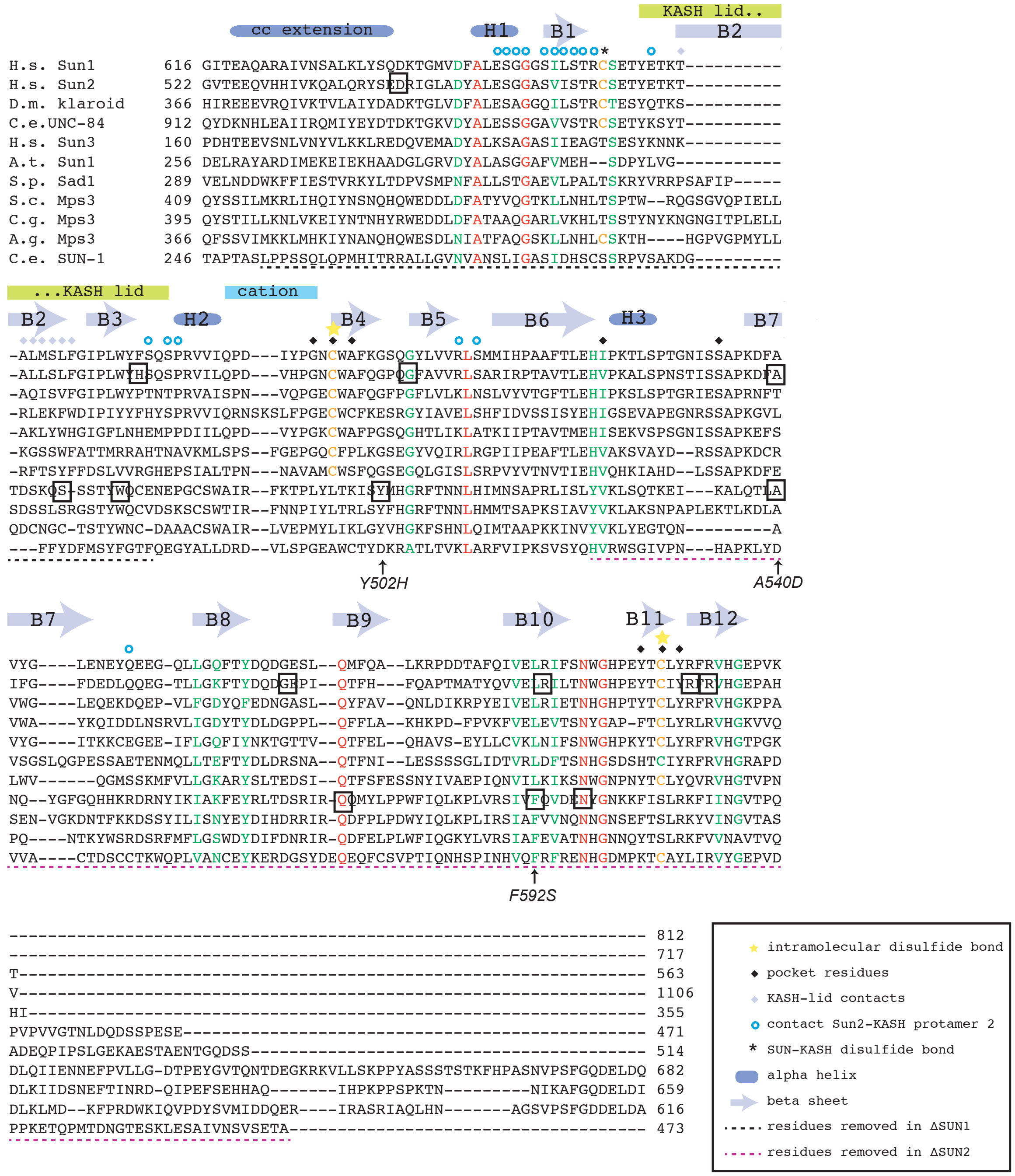

FIGURE S1. Sequence alignment of SUN domains. The indicated SUN domain sequences were aligned using ClustalW2. Conserved residues are shown in red and similar residues in green. Cysteine residues thought to participate in intramolecular disulfide bond and in formation of a disulfide bond with the KASH domain are highlighted in yellow (SOSA et al. 2012; ZHOU et al. 2012). These residues do not appear to be conserved in the plant or fungal SUN domains. Secondary structure elements are shown above the aligned sequences and are based on crystallographic data from human Sun2, which was also used to predict residues involved in association with the KASH domain or in formation of the SUN domain pocket (SOSA et al. 2012; ZHOU et al. 2012). Boxed residues indicate key residues that have been shown by mutagenesis to affect SUN-KASH binding or SUN protein function in vivo, including the three *mps3* alleles described (JASPERSEN et al. 2002; JASPERSEN et al. 2006; MALONE et al. 1999; NISHIKAWA et al. 2003; SOSA et al. 2012; ZHOU et al. 2012). The black and magenta dashed lines indicate the regions deleted in *mps3ΔSUN1* (415-480) and *mps3ΔSUN2* (524-645), respectively.

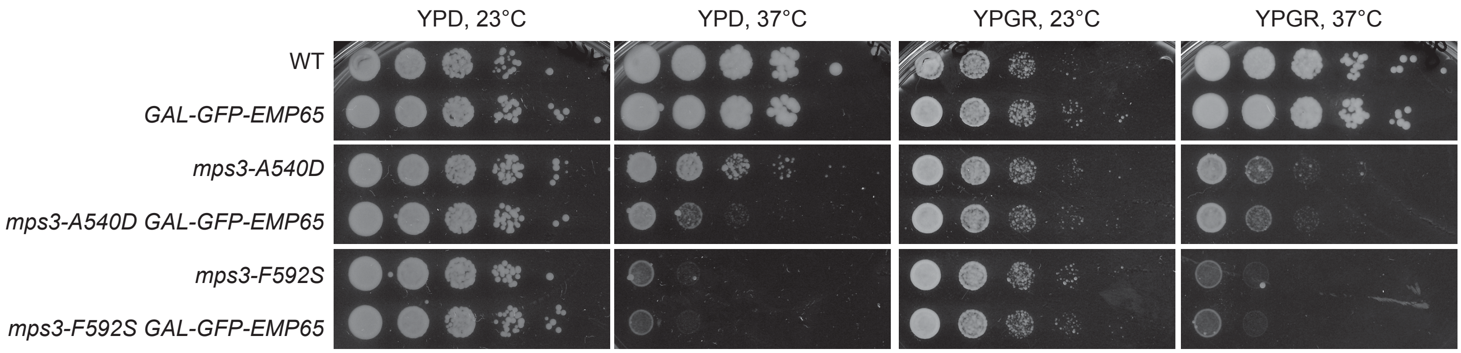

FIGURE S2. GAL-EMP65 does not affect cell growth. Wild-type (SLJ771), *mps3-A540D* (SLJ1622) and *mps3-F592S* (SLJ1711) cells containing *EMP65* expressed from the endogenous promoter and *GAL-GFP-EMP65* (SLJ4074), *GAL-GFP-EMP65 mps3-A540D* (SLJ5982) and *GAL-GFP-EMP65 mps3-F592S* (SLJ5985) containing *EMP65* expressed under the *GAL1* promoter were serially-diluted 10-fold and spotted onto YPD and YPGR plates. Plates were incubated at 30°C and 37°C for 2 d and at 23°C for 3 d. Although *GAL-GFP-EMP65* does not affect cell growth, the fact that *GAL-GFP-EMP65 mps3-A540D* and *GAL-GFP-EMP65 mps3-F592S* cells are viable and display no obvious phenotype on YPD at 23°C also indicates that *EMP65* is expressed at a low level even under repressing conditions. This was confirmed by western blot analysis and by imaging (data not shown).

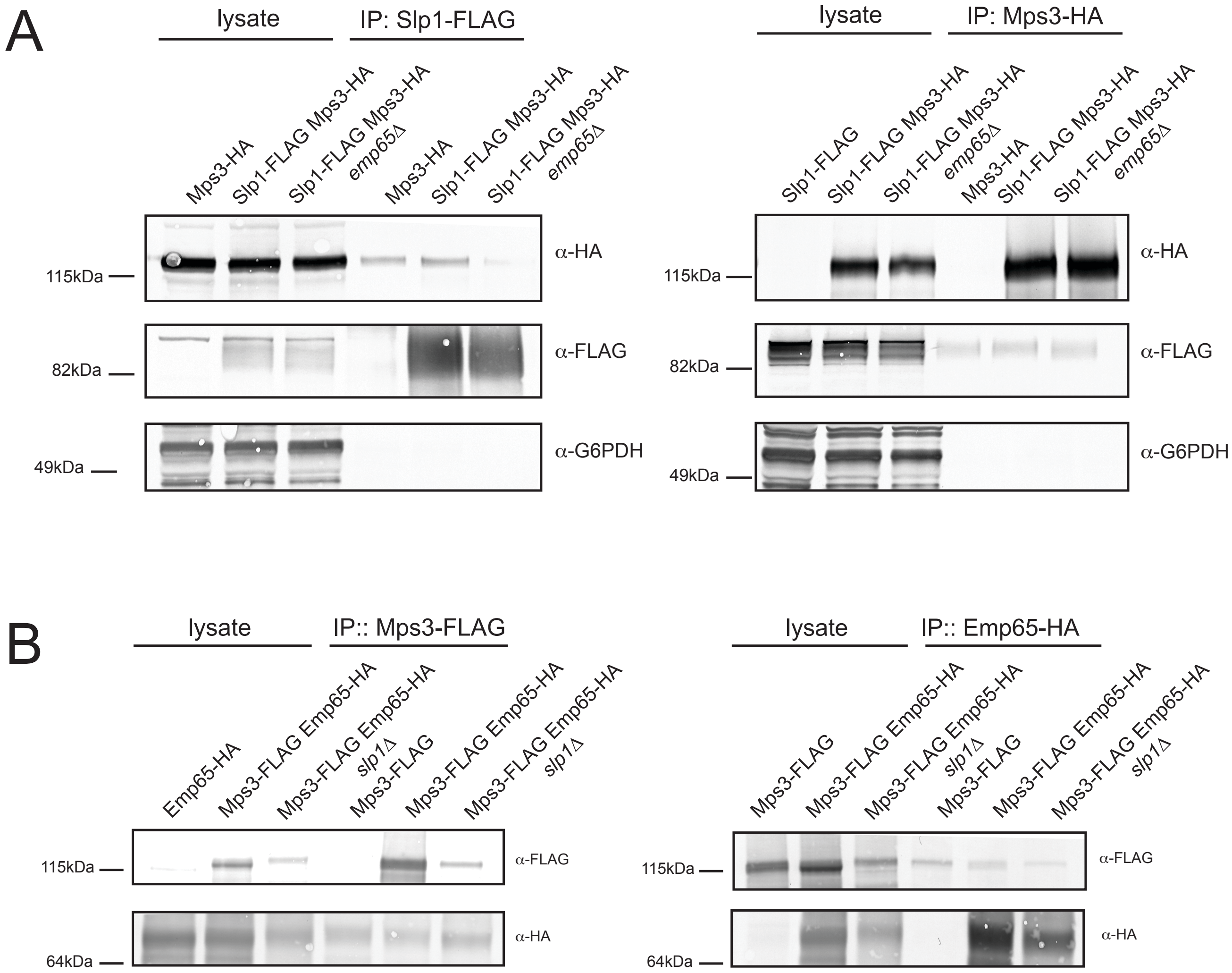

FIGURE S3. Mps3 does not co-immunoprecipitate with Slp1 or Emp65. A. Liquid nitrogen ground lysates were prepared from *MPS3-3xHA* (SLJ1234), *MPS3-3xHA SLP1-3xFLAG* (SLJ5854), *SLP1-3xFLAG* (SLJ3864) and *MPS3-3xHA SLP1-3xFLAG emp65Δ* (SLJ6151) strains. Proteins present in each lysate and bound to anti-FLAG beads or anti-HA beads were analyzed by immunoblotting with the indicated antibodies. B. Similarly, liquid nitrogen ground lysates were prepared from *MPS3-3xFLAG* (SLJ3529), *MPS3-3xFLAG 3xHA-EMP65* (SLJ5846), *3xHA-EMP65* (SLJ3837) and *MPS3-3xFLAG 3xHA-EMP65 slp1Δ* (SLJ6057) strains. Proteins present in each lysate and bound to anti-FLAG beads or anti-HA beads were analyzed by immunoblotting with the indicated antibodies. A-B. Positions of molecular weight markers are indicated on the left.

Table S1 Genetic interactions with *mps3* alleles.

Available for download as an Excel file at http://www.g3journal.org/lookup/suppl/doi:10.1534/g3.112.004614/-/DC1.

Table S2 Yeast strains used in this study

Strain	Background*	Relevant Genotype**	Experiment
SLJ1053	W303	*a mps3Δ::NATMX leu2-3,112 ADE2 lys2Δ pURA3-MPS3*	
SLJ1678	W303	*a mps3Δ::NATMX leu2::MPS3-LEU2 ADE2 lys2Δ pURA3-MPS3*	Figure 1A, 6B
SLJ1613	W303	*a mps3Δ::NATMX leu2::mps3ΔSUN1-LEU2 ADE2 lys2Δ pURA3-MPS3*	Figure 1A
SLJ1615 & SLJ1616	W303	*a mps3Δ::NATMX leu2::mps3ΔSUN2-LEU2 ADE2 lys2Δ pURA3-MPS3*	Figure 1A
SLJ1614	W303	*a mps3Δ::NATMX leu2::mps3ΔSUN-LEU2 ADE2 lys2Δ pURA3-MPS3*	Figure 1A
SLJ1712	W303	*a mps3Δ::HIS3MX leu2::mps3-Y502H-LEU2 ADE2 lys2Δ pURA3-MPS3*	Figure 1A, 7B
SLJ1622	W303	*a mps3Δ::NATMX leu2::mps3-A540D-LEU2 ADE2 lys2Δ pURA3-MPS3*	Figure 1A, 7B
SLJ1711	W303	*a mps3Δ::NATMX leu2::mps3-F592S-LEU2 ADE2 lys2Δ pURA3-MPS3*	Figure 1A, 7B, S2
SLJ1370	W303	*a mps3Δ::NATMX leu2::mps3Δ75-150-LEU2 ADE2 lys2Δ pURA3-MPS3*	Figure 1A
SLJ1883	BY	*α can1Δ::STE2pr-HIS5Sp lyp1Δ met15Δ0*	
SLJ4186	BY	*α mps3Δ::MPS3-NATMX can1Δ::STE2pr-HIS5Sp lyp1Δ met15Δ0 pURA3-MPS3*	Figure 1A
SLJ1885	BY	*α mps3Δ::mps3-Y502H-NATMX can1Δ::STE2pr-HIS5Sp lyp1Δ met15Δ0 pURA3-MPS3*	Figure 1A
SLJ1887	BY	*α mps3Δ::mps3-A540D-NATMX can1Δ::STE2pr-HIS5Sp lyp1Δ met15Δ0 pURA3-MPS3*	Figure 1A
SLJ1886	BY	*α mps3Δ::mps3-F592S-NATMX can1Δ::STE2pr-HIS5Sp lyp1Δ met15Δ0 pURA3-MPS3*	Figure 1A
SLJ2153	BY	*α mps3Δ::mps3Δ75-150-NATMX can1Δ::STE2pr-HIS5Sp lyp1Δ met15Δ0 pURA3-MPS3*	Figure 1A
SLJ6406	SK-1	*a mps3Δ::NATMX pURA3-MPS3*	
SLJ6407	SK-1	*α mps3Δ::NATMX pURA3-MPS3*	
SLJ6408 x SLJ6409	SK-1	*a/α mps3Δ::NATMX/mps3Δ::NATMX leu2::MPS3-LEU2/leu2::MPS3-LEU2 pURA3-MPS3*	Figure 1B, C
SLJ6417 x SLJ6418	SK-1	*a/α mps3Δ::NATMX/mps3Δ::NATMX leu2::mps3ΔSUN1-LEU2/leu2::mps3ΔSUN1-LEU2 pURA3-MPS3*	Figure 1B, C
SLJ6420 x SLJ6421	SK-1	*a/α mps3Δ::NATMX/mps3Δ::NATMX leu2::mps3ΔSUN2-LEU2/leu2::mps3ΔSUN2-LEU2 pURA3-MPS3*	Figure 1B, C
SLJ6423 x SLJ6424	SK-1	*a/α mps3Δ::NATMX/mps3Δ::NATMX leu2::mps3ΔSUN2-LEU2/leu2::mps3ΔSUN2-LEU2 pURA3-MPS3*	Figure 1B, C

SLJ6414 x SLJ6415	SK-1	*a/α mps3Δ::NATMX/mps3Δ::NATMX leu2::(2xmps3ΔSUN-LEU2)/leu2::(2xmps3ΔSUN-LEU2) pURA3-MPS3*	Figure 1B, C
SLJ6411 x SLJ6412	SK-1	*a/α mps3Δ::NATMX/mps3Δ::NATMX leu2::mps3Δ75-150-LEU2/leu2::mps3Δ75-150-LEU2 pURA3-MPS3*	Figure 1B, C
SLJ001	W303	*a*	Figure 5B, 5C, S2, S3A, S3B
SLJ3529	W303	*a mps3::MPS3-3xFLAG-KANMX*	Figure 5B, S3B
SLJ3864	W303	*a slp1::SLP1-3xFLAG-KANMX*	Figure 5B, 5C, S2, S3A
SLJ3837	W303	*a emp65Δ::GAL-3xHA-EMP65-HIS3MX*	Figure 5B, 5C, S2
SLJ4048	W303	*α slp1::SLP1-3xFLAG-KANMX emp65Δ::GAL-3xHA-EMP65-HIS3MX*	Figure 5C
SLJ6040	W303	*a mps3Δ::NATMX emp65Δ::GAL-3xHA-EMP65-HIS3MX pURA3-MPS3*	Figure 5D
SLJ6088	W303	*a mps3Δ::NATMX emp65Δ::GAL-3xHA-EMP65-HIS3MX SLP1-3xFLAG-KANMX leu2::MPS3-LEU2*	Figure 5D
SLJ6086	W303	*a mps3Δ::NATMX emp65Δ::GAL-3xHA-EMP65-HIS3MX SLP1-3xFLAG-KANMX leu2::mps3-F592S-LEU2*	Figure 5D
SLJ6087	W303	*a mps3Δ::NATMX emp65Δ::GAL-3xHA-EMP65-HIS3MX SLP1-3xFLAG-KANMX leu2::mps3-Y502H-LEU2*	Figure 5D
SLJ6092	W303	*a mps3Δ::NATMX emp65Δ::GAL-3xHA-EMP65-HIS3MX SLP1-3xFLAG-KANMX leu2::mps3Δ75-150-LEU2*	Figure 5D
SLJ6525	W303	*a slp1Δ::KANMX his3::SLP1-3xGFP-HIS3 trp1::HDEL-dsRED-TRP1 ADE2*	Figure 6A
SLJ4074	W303	*a emp65Δ::GAL-GFP-EMP65-KANMX trp1::HDEL-dsRED-TRP1 ADE2*	Figure 6B, S2
SLJ6430	BY	*α tub4::TUB4-GFP-HYGMX::mCherry-TUB1-URA3*	Figure 7A
SLJ6434	BY	*α slp1Δ::KANMX tub4::TUB4-GFP-HYGMX::mCherry-TUB1-URA3*	Figure 7A
SLJ6436	BY	*α emp65Δ::KANMX tub4::TUB4-GFP-HYGMX::mCherry-TUB1-URA3*	Figure 7A
SLJ3136	W303	*a slp1Δ::KANMX*	Figure 7B
SLJ3277	W303	*a emp65Δ::KANMX*	Figure 7B
	W303	*a htb2::HTB2-mCherry-URA3MX ADE2*	Figure 8B, C
	W303	*a mps3::MPS3-GFP-NATMX htb2::HTB2-mCherry-URA3MX ADE2 lys2Δ*	Figure 8A, B, C
	W303	*a slp1Δ::KANMX mps3::MPS3-GFP-NATMX htb2::HTB2-mCherry-URA3MX ADE2*	Figure 8A, B, C

	W303	*a emp65Δ::KANMX mps3::MPS3-GFP-NATMX htb2::HTB2-mCherry-URA3MX ADE2*	Figure 8A, B, C
SLJ5982	W303	*α mps3Δ::mps3-A540D emp65Δ::GAL-GFP-EMP65-KANMX*	Figure S2
SLJ5985	W303	*α mps3Δ::mps3-F592S emp65Δ::GAL-GFP-EMP65-KANMX*	Figure S2
SLJ771	W303	*a ADE2*	Figure S2
SLJ6086	W303	*a slp1::SLP1-3xFLAG-KANMX emp65Δ::GAL-3xHA-EMP65-HIS3MX mps3Δ::NATMX leu2::mps3-F592S-LEU2*	Figure S2
SLJ1234	W303	*α mps3::MPS3-3xHA-HIS3MX*	Figure S3A
SLJ5854	W303	*α mps3::MPS3-3xHA-HIS3MX slp1::SLP1-3xFLAG-KANMX*	Figure S3A
SLJ6151	W303	*α mps3::MPS3-3xHA-HIS3MX slp1::SLP1-3xFLAG-KANMX emp65Δ::HYGMX*	Figure S3A
SLJ5846	W303	*a mps3::MPS3-3xFLAG-KANMX emp65Δ::GAL-3xHA-EMP65-HIS3MX*	Figure S3B
SLJ6057	W303	*α mps3::MPS3-3xFLAG-KANMX emp65Δ::GAL-3xHA-EMP65-HIS3MX slp1Δ::NATMX*	Figure S3B

W303 are ade2-1 ura3-1 trp1-1 leu2-3,112 his3-11, 15 can1-100, BY are ura3Δ0 leu2Δ0 his3Δ1 and SK-1 are ho::hisG lys2 ura3 leu2::hisG trp1ΔFA::hisG his3-11,15

** pURA3-MPS3 was removed by plating cells to 5-FOA immediately before the experiment
