# Supplementary figures and images for: Genetic Analysis of Mps3 SUN Domain Mutants in Saccharomyces cerevisiae Reveals an Interaction with the SUN-Like Protein Slp1
Source: G3 (Bethesda). 2012 Dec 1;2(12):1703–18. doi: 10.1534/g3.112.004614 (PMC3516490; doi:10.1534/g3.112.004614)

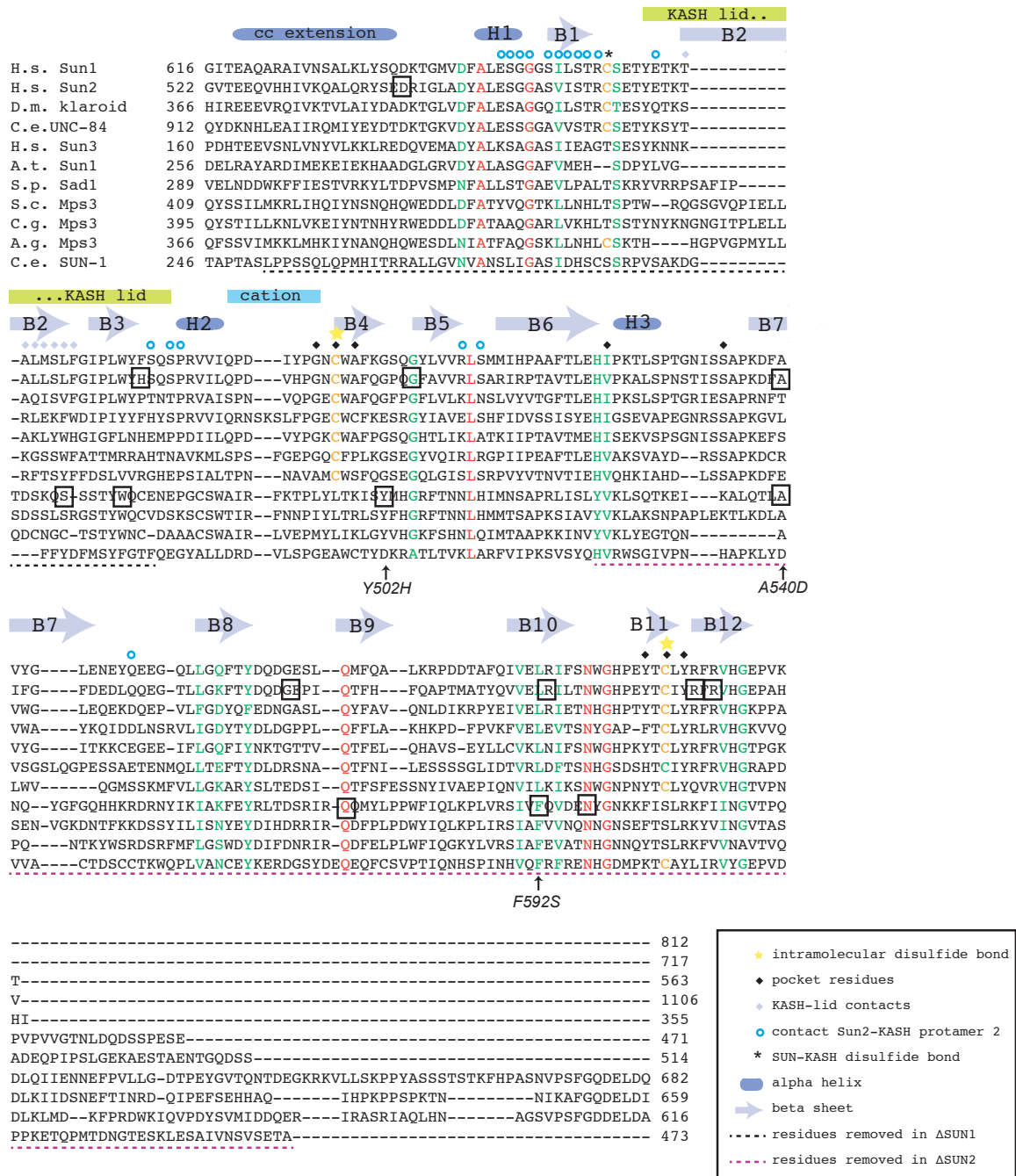

Supplement: Supporting Information [file supp_2.12.1703_FigureS1.pdf]
