# Supplementary material for: Genetic Analysis of Mps3 SUN Domain Mutants in Saccharomyces cerevisiae Reveals an Interaction with the SUN-Like Protein Slp1
Source: G3 (Bethesda). 2012 Dec 1;2(12):1703–18. doi: 10.1534/g3.112.004614 (PMC3516490; doi:10.1534/g3.112.004614)
Supplement: Supporting Information [file supp_2.12.1703_FigureS2.pdf]

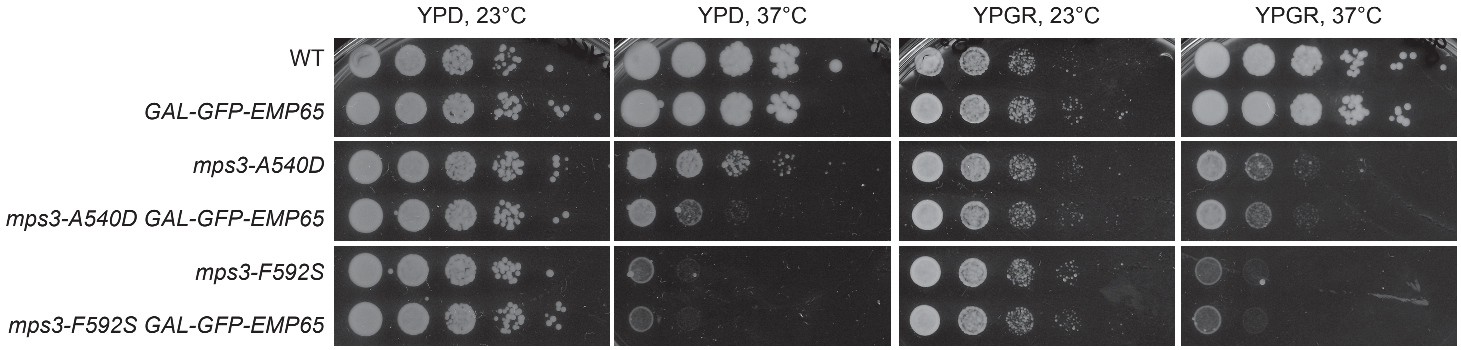

FIGURE S2. GAL-EMP65 does not affect cell growth. Wild-type (SLJ771), *mps3-A540D* (SLJ1622) and *mps3-F592S* (SLJ1711) cells containing *EMP65* expressed from the endogenous promoter and *GAL-GFP-EMP65* (SLJ4074), *GAL-GFP-EMP65 mps3-A540D* (SLJ5982) and *GAL-GFP-EMP65 mps3-F592S* (SLJ5985) containing *EMP65* expressed under the *GAL1* promoter were serially-diluted 10-fold and spotted onto YPD and YPGR plates. Plates were incubated at 30°C and 37°C for 2 d and at 23°C for 3 d. Although *GAL-GFP-EMP65* does not affect cell growth, the fact that *GAL-GFP-EMP65 mps3-A540D* and *GAL-GFP-EMP65 mps3-F592S* cells are viable and display no obvious phenotype on YPD at 23°C also indicates that *EMP65* is expressed at a low level even under repressing conditions. This was confirmed by western blot analysis and by imaging (data not shown).
