# Supplementary material for: Genetic Analysis of Mps3 SUN Domain Mutants in Saccharomyces cerevisiae Reveals an Interaction with the SUN-Like Protein Slp1
Source: G3 (Bethesda). 2012 Dec 1;2(12):1703–18. doi: 10.1534/g3.112.004614 (PMC3516490; doi:10.1534/g3.112.004614)
Supplement: Supporting Information [file supp_2.12.1703_FigureS3.pdf]

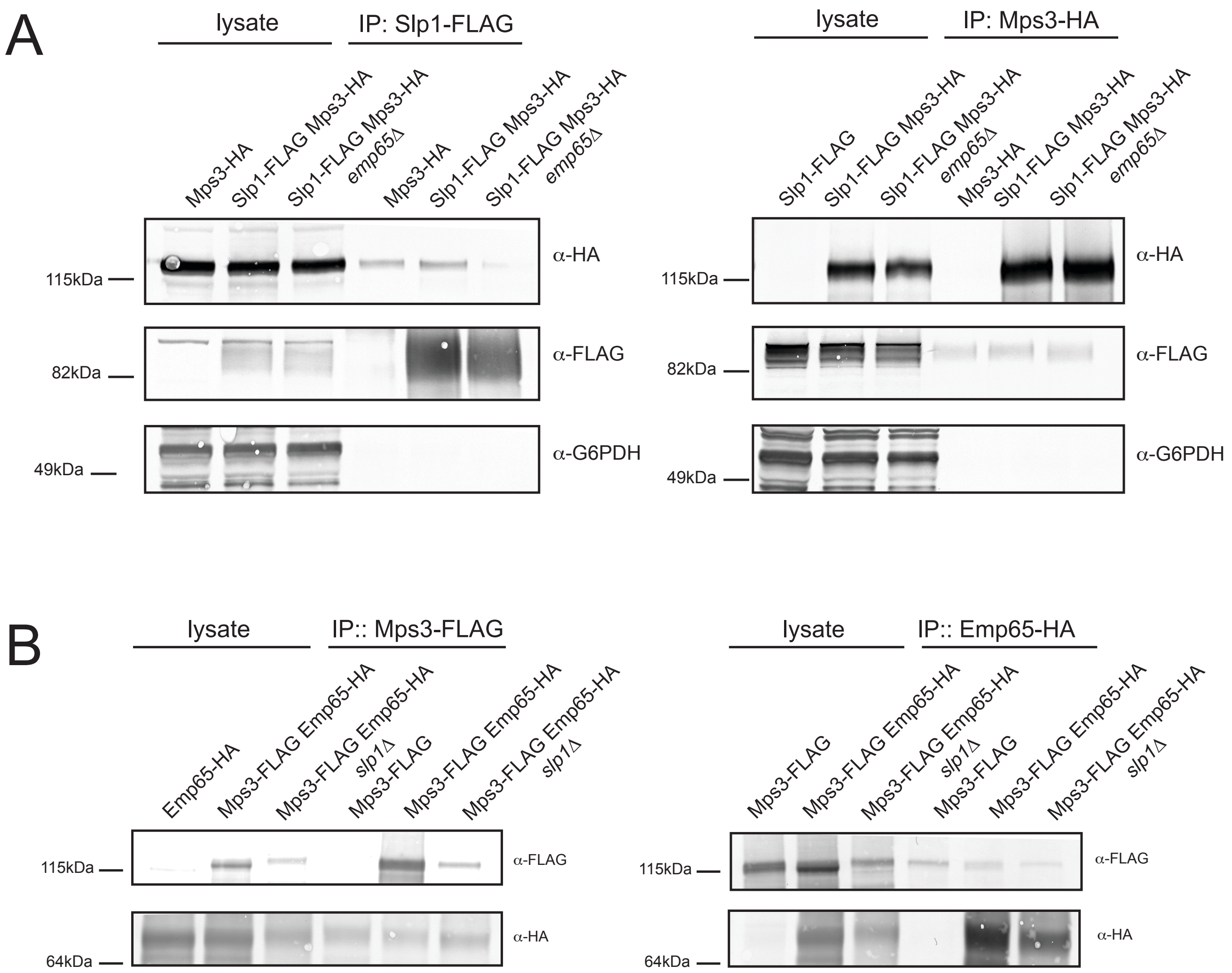

FIGURE S3. Mps3 does not co-immunoprecipitate with Slp1 or Emp65. A. Liquid nitrogen ground lysates were prepared from *MPS3-3xHA* (SLJ1234), *MPS3-3xHA SLP1-3xFLAG* (SLJ5854), *SLP1-3xFLAG* (SLJ3864) and *MPS3-3xHA SLP1-3xFLAG emp65Δ* (SLJ6151) strains. Proteins present in each lysate and bound to anti-FLAG beads or anti-HA beads were analyzed by immunoblotting with the indicated antibodies. B. Similarly, liquid nitrogen ground lysates were prepared from *MPS3-3xFLAG* (SLJ3529), *MPS3-3xFLAG 3xHA-EMP65* (SLJ5846), *3xHA-EMP65* (SLJ3837) and *MPS3-3xFLAG 3xHA-EMP65 slp1Δ* (SLJ6057) strains. Proteins present in each lysate and bound to anti-FLAG beads or anti-HA beads were analyzed by immunoblotting with the indicated antibodies. A-B. Positions of molecular weight markers are indicated on the left.
