# Supplementary material for: Genetic Analysis of Mps3 SUN Domain Mutants in Saccharomyces cerevisiae Reveals an Interaction with the SUN-Like Protein Slp1
Source: G3 (Bethesda). 2012 Dec 1;2(12):1703–18. doi: 10.1534/g3.112.004614 (PMC3516490; doi:10.1534/g3.112.004614)
Supplement: Supporting Information [file supp_2.12.1703_TableS2.pdf]

**Table S2 Yeast strains used in this study**

| Strain | Background* | Relevant Genotype** | Experiment |
|---|---|---|---|
| SLJ1053 | W303 | *a mps3Δ::NATMX leu2-3,112 ADE2 lys2Δ pURA3-MPS3* | |
| SLJ1678 | W303 | *a mps3Δ::NATMX leu2::MPS3-LEU2 ADE2 lys2Δ pURA3-MPS3* | Figure 1A, 6B |
| SLJ1613 | W303 | *a mps3Δ::NATMX leu2::mps3ΔSUN1-LEU2 ADE2 lys2Δ pURA3-MPS3* | Figure 1A |
| SLJ1615 & SLJ1616 | W303 | *a mps3Δ::NATMX leu2::mps3ΔSUN2-LEU2 ADE2 lys2Δ pURA3-MPS3* | Figure 1A |
| SLJ1614 | W303 | *a mps3Δ::NATMX leu2::mps3ΔSUN-LEU2 ADE2 lys2Δ pURA3-MPS3* | Figure 1A |
| SLJ1712 | W303 | *a mps3Δ::HIS3MX leu2::mps3-Y502H-LEU2 ADE2 lys2Δ pURA3-MPS3* | Figure 1A, 7B |
| SLJ1622 | W303 | *a mps3Δ::NATMX leu2::mps3-A540D-LEU2 ADE2 lys2Δ pURA3-MPS3* | Figure 1A, 7B |
| SLJ1711 | W303 | *a mps3Δ::NATMX leu2::mps3-F592S-LEU2 ADE2 lys2Δ pURA3-MPS3* | Figure 1A, 7B, S2 |
| SLJ1370 | W303 | *a mps3Δ::NATMX leu2::mps3Δ75-150-LEU2 ADE2 lys2Δ pURA3-MPS3* | Figure 1A |
| SLJ1883 | BY | *α can1Δ::STE2pr-HIS5Sp lyp1Δ met15Δ0* | |
| SLJ4186 | BY | *α mps3Δ::MPS3-NATMX can1Δ::STE2pr-HIS5Sp lyp1Δ met15Δ0 pURA3-MPS3* | Figure 1A |
| SLJ1885 | BY | *α mps3Δ::mps3-Y502H-NATMX can1Δ::STE2pr-HIS5Sp lyp1Δ met15Δ0 pURA3-MPS3* | Figure 1A |
| SLJ1887 | BY | *α mps3Δ::mps3-A540D-NATMX can1Δ::STE2pr-HIS5Sp lyp1Δ met15Δ0 pURA3-MPS3* | Figure 1A |
| SLJ1886 | BY | *α mps3Δ::mps3-F592S-NATMX can1Δ::STE2pr-HIS5Sp lyp1Δ met15Δ0 pURA3-MPS3* | Figure 1A |
| SLJ2153 | BY | *α mps3Δ::mps3Δ75-150-NATMX can1Δ::STE2pr-HIS5Sp lyp1Δ met15Δ0 pURA3-MPS3* | Figure 1A |
| SLJ6406 | SK-1 | *a mps3Δ::NATMX pURA3-MPS3* | |
| SLJ6407 | SK-1 | *α mps3Δ::NATMX pURA3-MPS3* | |
| SLJ6408 x SLJ6409 | SK-1 | *a/α mps3Δ::NATMX/mps3Δ::NATMX leu2::MPS3-LEU2/leu2::MPS3-LEU2 pURA3-MPS3* | Figure 1B, C |
| SLJ6417 x SLJ6418 | SK-1 | *a/α mps3Δ::NATMX/mps3Δ::NATMX leu2::mps3ΔSUN1-LEU2/leu2::mps3ΔSUN1-LEU2 pURA3-MPS3* | Figure 1B, C |
| SLJ6420 x SLJ6421 | SK-1 | *a/α mps3Δ::NATMX/mps3Δ::NATMX leu2::mps3ΔSUN2-LEU2/leu2::mps3ΔSUN2-LEU2 pURA3-MPS3* | Figure 1B, C |
| SLJ6423 x SLJ6424 | SK-1 | *a/α mps3Δ::NATMX/mps3Δ::NATMX leu2::mps3ΔSUN2-LEU2/leu2::mps3ΔSUN2-LEU2 pURA3-MPS3* | Figure 1B, C |

| | | | |
|---|---|---|---|
| SLJ6414 x SLJ6415 | SK-1 | *a/α mps3Δ::NATMX/mps3Δ::NATMX leu2::(2xmps3ΔSUN-LEU2)/leu2::(2xmps3ΔSUN-LEU2) pURA3-MPS3* | Figure 1B, C |
| SLJ6411 x SLJ6412 | SK-1 | *a/α mps3Δ::NATMX/mps3Δ::NATMX leu2::mps3Δ75-150-LEU2/leu2::mps3Δ75-150-LEU2 pURA3-MPS3* | Figure 1B, C |
| SLJ001 | W303 | *a* | Figure 5B, 5C, S2, S3A, S3B |
| SLJ3529 | W303 | *a mps3::MPS3-3xFLAG-KANMX* | Figure 5B, S3B |
| SLJ3864 | W303 | *a slp1::SLP1-3xFLAG-KANMX* | Figure 5B, 5C, S2, S3A |
| SLJ3837 | W303 | *a emp65Δ::GAL-3xHA-EMP65-HIS3MX* | Figure 5B, 5C, S2 |
| SLJ4048 | W303 | *α slp1::SLP1-3xFLAG-KANMX emp65Δ::GAL-3xHA-EMP65-HIS3MX* | Figure 5C |
| SLJ6040 | W303 | *a mps3Δ::NATMX emp65Δ::GAL-3xHA-EMP65-HIS3MX pURA3-MPS3* | Figure 5D |
| SLJ6088 | W303 | *a mps3Δ::NATMX emp65Δ::GAL-3xHA-EMP65-HIS3MX SLP1-3xFLAG-KANMX leu2::MPS3-LEU2* | Figure 5D |
| SLJ6086 | W303 | *a mps3Δ::NATMX emp65Δ::GAL-3xHA-EMP65-HIS3MX SLP1-3xFLAG-KANMX leu2::mps3-F592S-LEU2* | Figure 5D |
| SLJ6087 | W303 | *a mps3Δ::NATMX emp65Δ::GAL-3xHA-EMP65-HIS3MX SLP1-3xFLAG-KANMX leu2::mps3-Y502H-LEU2* | Figure 5D |
| SLJ6092 | W303 | *a mps3Δ::NATMX emp65Δ::GAL-3xHA-EMP65-HIS3MX SLP1-3xFLAG-KANMX leu2::mps3Δ75-150-LEU2* | Figure 5D |
| SLJ6525 | W303 | *a slp1Δ::KANMX his3::SLP1-3xGFP-HIS3 trp1::HDEL-dsRED-TRP1 ADE2* | Figure 6A |
| SLJ4074 | W303 | *a emp65Δ::GAL-GFP-EMP65-KANMX trp1::HDEL-dsRED-TRP1 ADE2* | Figure 6B, S2 |
| SLJ6430 | BY | *α tub4::TUB4-GFP-HYGMX::mCherry-TUB1-URA3* | Figure 7A |
| SLJ6434 | BY | *α slp1Δ::KANMX tub4::TUB4-GFP-HYGMX::mCherry-TUB1-URA3* | Figure 7A |
| SLJ6436 | BY | *α emp65Δ::KANMX tub4::TUB4-GFP-HYGMX::mCherry-TUB1-URA3* | Figure 7A |
| SLJ3136 | W303 | *a slp1Δ::KANMX* | Figure 7B |
| SLJ3277 | W303 | *a emp65Δ::KANMX* | Figure 7B |
| | W303 | *a htb2::HTB2-mCherry-URA3MX ADE2* | Figure 8B, C |
| | W303 | *a mps3::MPS3-GFP-NATMX htb2::HTB2-mCherry-URA3MX ADE2 lys2Δ* | Figure 8A, B, C |
| | W303 | *a slp1Δ::KANMX mps3::MPS3-GFP-NATMX htb2::HTB2-mCherry-URA3MX ADE2* | Figure 8A, B, C |

| | | | |
|---|---|---|---|
| | W303 | *a emp65Δ::KANMX mps3::MPS3-GFP-NATMX htb2::HTB2-mCherry-URA3MX ADE2* | Figure 8A, B, C |
| SLJ5982 | W303 | *α mps3Δ::mps3-A540D emp65Δ::GAL-GFP-EMP65-KANMX* | Figure S2 |
| SLJ5985 | W303 | *α mps3Δ::mps3-F592S emp65Δ::GAL-GFP-EMP65-KANMX* | Figure S2 |
| SLJ771 | W303 | *a ADE2* | Figure S2 |
| SLJ6086 | W303 | *a slp1::SLP1-3xFLAG-KANMX emp65Δ::GAL-3xHA-EMP65-HIS3MX mps3Δ::NATMX leu2::mps3-F592S-LEU2* | Figure S2 |
| SLJ1234 | W303 | *α mps3::MPS3-3xHA-HIS3MX* | Figure S3A |
| SLJ5854 | W303 | *α mps3::MPS3-3xHA-HIS3MX slp1::SLP1-3xFLAG-KANMX* | Figure S3A |
| SLJ6151 | W303 | *α mps3::MPS3-3xHA-HIS3MX slp1::SLP1-3xFLAG-KANMX emp65Δ::HYGMX* | Figure S3A |
| SLJ5846 | W303 | *a mps3::MPS3-3xFLAG-KANMX emp65Δ::GAL-3xHA-EMP65-HIS3MX* | Figure S3B |
| SLJ6057 | W303 | *α mps3::MPS3-3xFLAG-KANMX emp65Δ::GAL-3xHA-EMP65-HIS3MX slp1Δ::NATMX* | Figure S3B |

W303 are ade2-1 ura3-1 trp1-1 leu2-3,112 his3-11, 15 can1-100, BY are ura3Δ0 leu2Δ0 his3Δ1 and SK-1 are ho::hisG lys2 ura3 leu2::hisG trp1ΔFA::hisG his3-11,15

** pURA3-MPS3 was removed by plating cells to 5-FOA immediately before the experiment
